# Fructose promotes angiogenesis by improving vascular endothelial cell function and upregulating VEGF expression in cancer cells

**DOI:** 10.1186/s13046-023-02765-3

**Published:** 2023-07-28

**Authors:** Yanfen Cui, Hui Liu, Zhaosong Wang, He Zhang, Jianfei Tian, Zhiyong Wang, Weijie Song, Hui Guo, Liming Liu, Ruinan Tian, Xiaoyan Zuo, Sixin Ren, Fei Zhang, Ruifang Niu

**Affiliations:** 1 Public Laboratory, Key Laboratory of Cancer Prevention and Therapy, Tianjin Medical University Cancer Institute and Hospital, National Clinical Research Center for Cancer, Tianjin’s Clinical Research Center for Cancer, Tianjin, 300060 China; 2 Laboratory Animal Center, Key Laboratory of Cancer Prevention and Therapy, Tianjin Medical University Cancer Institute and Hospital, National Clinical Research Center for Cancer, Tianjin’s Clinical Research Center for Cancer, Tianjin, 300060 China

**Keywords:** Fructose, Vascular Endothelial Cell, Angiogenesis, VEGF, Colorectal Cancer

## Abstract

**Background:**

Fructose is a very common sugar found in natural foods, while current studies demonstrate that high fructose intake is significantly associated with increased risk of multiple cancers and more aggressive tumor behavior, but the relevant mechanisms are not fully understood.

**Methods:**

Tumor-grafting experiments and in vitro angiogenesis assays were conducted to detect the effect of fructose and the conditioned medium of fructose-cultured tumor cells on biological function of vascular endothelial cells (VECs) and angiogenesis. 448 colorectal cancer specimens were utilized to analyze the relationship between Glut5 expression levels in VECs and tumor cells and microvascular density (MVD).

**Results:**

We found that fructose can be metabolized by VECs and activate the Akt and Src signaling pathways, thereby enhancing the proliferation, migration, and tube-forming abilities of VECs and thereby promoting angiogenesis. Moreover, fructose can also improve the expression of vascular endothelial growth factor (VEGF) by upregulating the production of reactive oxygen species (ROS) in colorectal cancer cells, thus indirectly enhancing the biological function of VECs. Furthermore, this pro-angiogenic effect of fructose metabolism has also been well validated in clinical colorectal cancer tissues and mouse models. Fructose contributes to angiogenesis in mouse subcutaneous tumor grafts, and MVD is positively correlated with Glut5 expression levels of both endothelial cells and tumor cells of human colorectal cancer specimens.

**Conclusions:**

These findings establish the direct role and mechanism by which fructose promotes tumor progression through increased angiogenesis, and provide reliable evidence for a better understanding of tumor metabolic reprogramming.

**Supplementary Information:**

The online version contains supplementary material available at 10.1186/s13046-023-02765-3.

## Background

Fructose, an isomer of glucose, is abundant in fruits and honey in an unbound state. Due to its high sweetness and low price, fructose has been widely used as a sweetener in daily foods. With the introduction of high fructose corn syrup (HFCS) to the food processing industry in the 1970s, fructose consumption increased significantly annually [1]. However, the results of large-scale epidemiological studies and in vivo animal experiments have shown that high fructose intake not only causes metabolic syndromes such as obesity and non-alcoholic fatty liver disease, but is also strongly related to the occurrence and development of multiple tumors [2–6]. Moreover, fructose can also promote the occurrence and progression of intestinal tumors in mice, even in the absence of obesity and metabolic syndrome [7]. In addition, high fructose intake causes deterioration of the intestinal barrier and subsequent endotoxemia, while constant inflammatory irritation eventually leads to liver cancer [8]. As mentioned above, research on the relationship between fructose intake and cancer has revealed some startling findings. Therefore, a deeper understanding of the mechanism by which fructose contributes to the malignant progression of cancer will provide a crucial basis for more effective cancer prevention and treatment.

Fructose is generally thought to be metabolized in only a few tissues, such as the liver, adipose tissue, and small intestine [9]. However, in recent years, researchers have found that fructose can also be directly metabolized by tumor cells as an alternative carbon source [10–13]. Meanwhile, glucose transporter 5 (Glut5, encoded by *SLC2A5*), a specific transporter of fructose, was found to be upregulated in these tumor cells, and its expression level was closely correlated with malignant evolution and clinical prognosis [12, 14, 15]. Studies have revealed that fructose metabolism can preferentially increase nucleic acid synthesis through the non-oxidized pentose phosphate pathway to promote pancreatic cancer growth [10], and increase the flux of the serine synthesis pathway to contribute to the proliferation of acute myeloid leukemia cells [16]. In addition to providing a carbon source for tumor cells, fructose can also improve the utilization of glutamine by leukemia cells [16]. Moreover, fructose regulates the activation of the AMPK/mTORC1 signaling pathway, thereby promoting the proliferation of lung cancer cells [15]. In addition, fructose also promotes breast cancer migration by increasing the level of glycosylation of cell surface proteins [17]. Thus, fructose is involved in regulating tumor progression through a variety of mechanisms in different tumors.

Blood vessels are critical for tumor survival and malignant progression [18]. Angiogenesis, the generation of new blood vessels from the existing vasculature, is a complex process involving the antagonistic effects of multiple pro- and anti-angiogenic factors [19]. More specifically, angiogenesis involves the proliferation, migration and structural rearrangement of endothelial cells, a process that requires energy and biomass [20–22]. The metabolism of vascular endothelial cells (VECs) is the engine that drives angiogenesis, suggesting a critical role of VEC metabolism in angiogenesis [23]. Generally, there is a monolayer of VECs in the quiescent vasculature. Upon exposure to external stimuli, such as hypoxia or nutrient changes, quiescent VECs can rapidly transition to an angiogenic state [24]. For tumors, increased angiogenesis can provide more energy and nutrients for tumor growth, and angiogenesis itself is an energy-demanding process [25–27]. The metabolic evolution of tumors allows them to utilize several nutrients that normal cells cannot metabolize, such as fructose, to meet their rapid proliferation needs. However, whether fructose, an important carbon source in the blood, can be used by VECs to accelerate angiogenesis has not been reported. In this study, we investigated the biochemical and molecular mechanisms involved in the promotion of angiogenesis by fructose. Our findings provide novel insights to better understand the role of fructose in regulating the overall metabolic reprogramming of tumors.

## Results

### Fructose contributes to angiogenesis in subcutaneous tumor grafts in mice

**Fig. 1:**
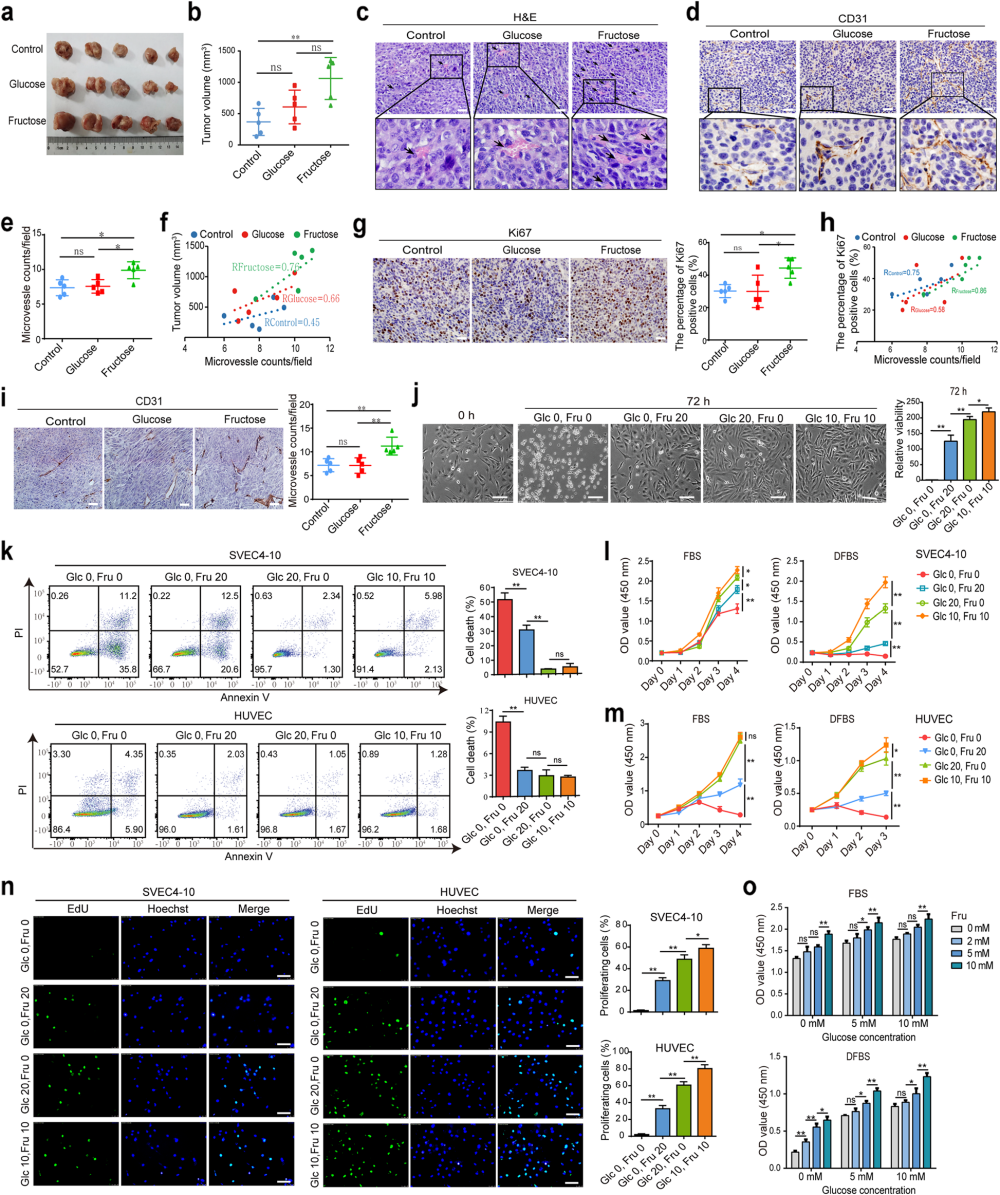
Fructose promotes tumor angiogenesis in vivo and VEC viability in vitro*.* **a**, Images of CT26.WT subcutaneous tumors in the indicated groups of mice (5 mice per group). **b**, Tumor volumes of mice. **c** and **d**, Representative images of H&E staining and CD31 IHC staining of the dissected tumors. Black arrows indicate the vessels in H&E stained images. Scale bar: 20 μm. **e**, MVDs counted by CD31 staining in mouse tumors. 10 fields (400 ×) within hotspot areas with higher MVD were examined. Scale bar: 20 μm. **f**, Linear relationship between MVD and tumor volume. **g**, Ki67-positive index in tumor tissues of each group detected by IHC staining. The percentages of Ki67-positive cells were calculated. Scale bar: 20 μm. **h**, Linear relationship between MVD and Ki67 positive index in tumors. **i**, CD31 staining and MVDs in Panc02 subcutaneous graft tumors in three groups of mice (*n* = 5 mice per group). Scale bar: 20 μm. **j**, SVEC4-10 cells were cultured in the four kinds of media containing 10% dialyzed FBS (DFBS) for 72 h. Photographs were taken at 0 h and 72 h, and the cell viability was detected by viable cell counts. Scale bar: 100 μm. **k**, Cell death of SVEC4-10 and HUVEC cultured under the above four kinds of media for 48 h. **l** and **m**, CCK-8 analysis was used to detect the proliferation of SVEC4-10 (**l**) and HUVEC (**m**) cells under the four media containing 10% FBS or DFBS. **n**, Images and quantitative analysis of EdU staining in SVEC4-10 and HUVEC cells cultured under the four conditions containing 10% DFBS for 48 h. Scale bar: 50 μm. **o**, The effect of elevated fructose concentrations on SVEC4-10 cell viability under specific glucose concentrations was analyzed using CCK-8. All data are expressed as the mean ± SD; ns, non-significant; *p < 0.05; **p < 0.01

Fructose is thought to contribute to the occurrence and progression of intestinal cancer in mice [7]. To further verify the effect of fructose on colorectal cancer and explore its specific mechanism, we established a tumor-bearing mouse model using the CT26.WT cell line. Then, all mice were randomly divided into three groups and fed different drinking water, blank water (control group), 10% glucose water (glucose group) and 10% fructose water (fructose group). Daily water/food intake and body weight were not significantly different among the three groups of mice. However, compared with the control group, both fructose and glucose promoted tumor growth in vivo, and the effect of fructose was more remarkable (Fig. 1a and 1b). Importantly, H&E staining (Fig. 1c) and CD31 IHC analysis (Fig. 1d and 1e) revealed that the microvascular density (MVD) of tumor tissues was significantly increased in the fructose group, and displayed a stronger positive correlation with tumor volume (Fig. 1f). In addition, the Ki67 index was higher in the fructose group (Fig. 1g), and the MVD was positively correlated with the Ki67 index (Fig. 1h). Consistent with these findings, similar results were obtained in another mouse model using the MC38 colon cancer cell line (Fig. S1a-g). These results strongly indicate that fructose accelerates tumor growth in vivo, at least in part due to the angiogenesis-promoting effect of fructose on the tumor. This possibility was also confirmed in a subcutaneous graft model based on the mouse pancreatic cancer cell line Panc02, using the same feeding conditions as described above. Gross examination and CD31 staining results showed that tumors in the fructose group were also significantly vascularized (Fig. 1i). Taken together, these results suggest a strong association between high daily fructose intake and tumor angiogenesis in vivo.

### Fructose promotes VEC proliferation and inhibits apoptosis

To investigate the effect of fructose on VEC viability in vitro, the vascular endothelial cell lines SVEC4-10 and HUVEC were cultured in four kinds of media, sugar-free medium (without fructose and glucose, named Glc 0, Fru 0), glucose medium (20 mM glucose only, named Glc 20, Fru 0), fructose medium (20 mM fructose only, named Glc 0, Fru 20), and double-sugar medium (10 mM fructose and 10 mM glucose, named Glc 10, Fru 10), in the presence of 10% FBS or dialyzed FBS (DFBS). After 72 h of culture in the above media, SVEC4-10 cells were able to survive and proliferate in the fructose medium, but not in the sugar-free medium (Fig. 1j). At the same concentration, the viability of SVEC4-10 in fructose medium was weaker than that in glucose medium, while the viability in double-sugar medium was the strongest (Figure 1j). Meanwhile, the flow cytometry results showed that fructose greatly reduced cell death in SVEC4-10 and HUVEC cells as detected by Annexin V-PI double staining (Fig. 1k). Furthermore, CCK-8 analysis showed that fructose promoted the proliferation of VECs, and the highest proliferation rate was observed in double-sugar medium (Fig. 1l and 1m), which was consistent with the results of cell counting by trypan blue staining (Fig. S1h and 1i). Interestingly, after 2 weeks of culture in fructose medium, the proliferation ability of SVEC4-10 cells was significantly increased to almost or even higher than that in glucose medium (Fig. S2a and S2b), indicating that long-term exposure to fructose can alter the biological behavior of VECs. Moreover, EdU (5-Ethynyl-2’-deoxyuridine) experiment (Fig. 1n) and Ki67 staining (Fig. S1j) also indicated that fructose significantly contributed to the proliferation of VECs. In addition, fructose significantly enhanced the viability of SVEC4-10 cells in a concentration-dependent manner under distinct glucose conditions, such as glucose deficiency (0 mM), glucose physiology (5 mM), or glucose sufficiency (10 mM) (Fig. 1o). Considering that hypoxia is a common phenomenon in solid tumors [28], we further investigated the effect of fructose on the biological behavior of VECs under hypoxic conditions, and found that fructose also boosted cell viability (Fig. S3a) and inhibited cell apoptosis (Fig. S3b). In conclusion, these results convincingly suggest that fructose promotes VEC proliferation and inhibits apoptosis in vitro.

### Fructose is absorbed and metabolized by VECs and increases cell viability

**Fig. 2:**
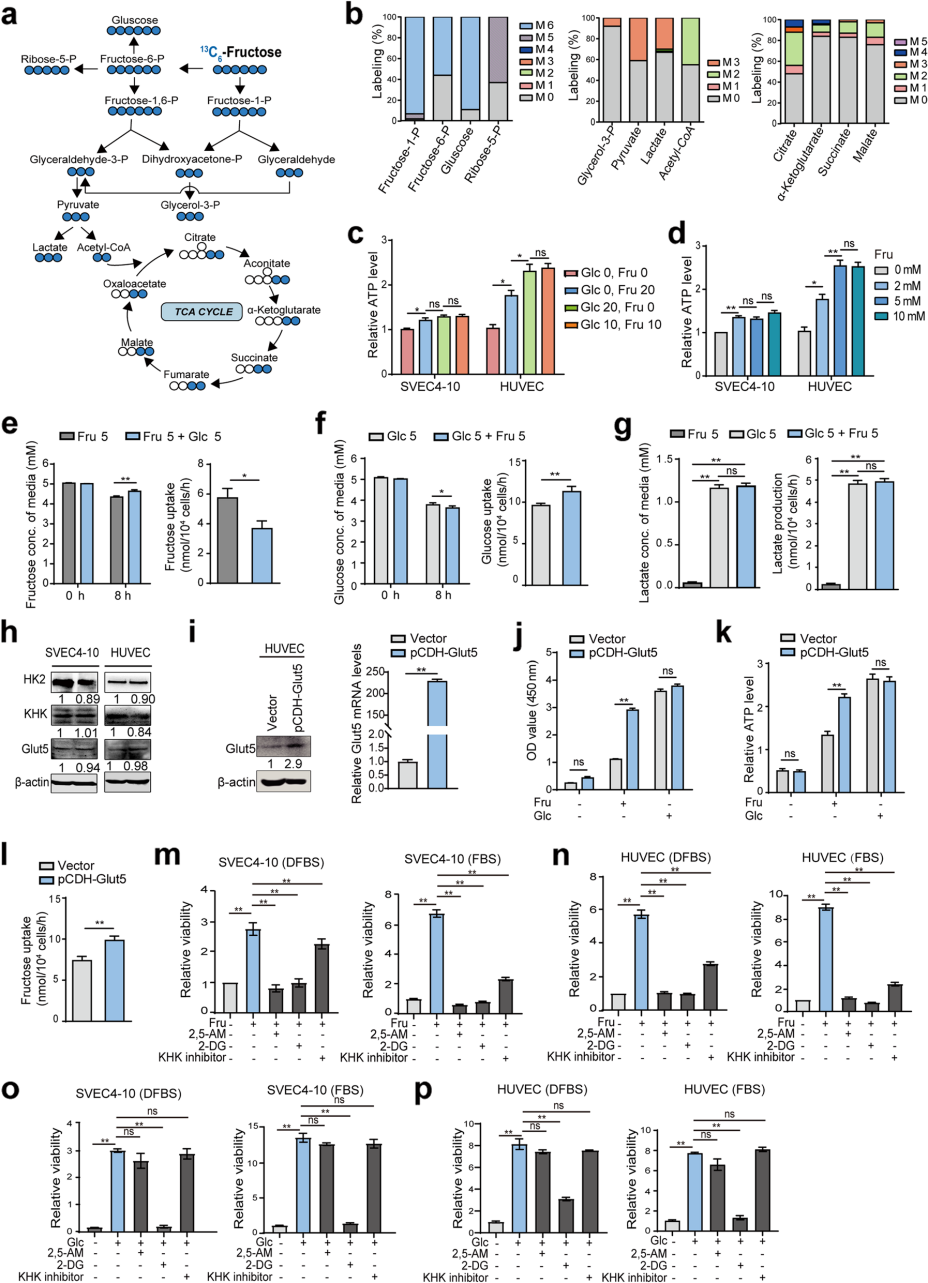
Fructose is absorbed and metabolized by VECs and increases cell viability. **a**, SVEC4-10 cells were incubated with 5 mM [U-13C] fructose in glucose-free medium for 8 h, and the intracellular metabolites were analyzed by LC/MS. Carbon isotope traces are shown, with blue circles indicating 13C-labeled carbon in intermediate metabolites. **b**, the percentage of corresponding isotope (e.g. pyruvate M + 3) in all isotopes (e.g. pyruvate: M + 0, M + 1, M + 2, M + 3) was calculated and corrected for natural isotopic abundance. **c**, Relative ATP levels of SVEC4-10 and HUVEC cells cultured in different media for 8 h. **d**, Relative ATP levels of SVEC4-10 and HUVEC cells cultured in various fructose concentrations for 8 h. **e**, Fructose concentration and consumption rates of SVEC4-10 cells in double sugar medium (Fru 5 mM + Glc 5 mM) and fructose medium (Fru 5 mM). **f**, Glucose concentration and consumption rates of SVEC4-10 cells in double sugar medium (Fru 5 mM + Glc 5 mM) and glucose medium (Glc 5 mM). **g**, Extracellular lactate concentrations and production rates of SVEC4-10 cultured in different media for 8 h. **h**, Western blot analysis of fructose metabolism-related protein expression levels in SVEC4-10 and HUVEC cells. **i**, Protein and mRNA expression levels of Glut5 in HUVEC cells overexpressing Glut5. **j**, Viability of HUVEC cells overexpressing Glut5 cultured in different media for 48 h. **k**, Relative ATP levels of HUVEC cells overexpressing Glut5 cultured in different media for 8 h. **l**, Effect of Glut5 overexpression on fructose consumption in HUVEC cells cultured in fructose medium. **m**-**p**, CCK-8 analysis of the viability of SVEC4-10 and HUVEC cells cultured for 48 h in medium containing 20 mM fructose (**m**, **n**) or glucose (**o**, **p**) in the presence of 2, 5-AM (3 mM), 2-DG (2 mM), and KHK inhibitor (1 μM), respectively. All data are expressed as the mean ± SD; ns, non-significant; *p < 0.05; **p < 0.01. *n* = 3

Having established that fructose affects the viability of VECs, we next examined whether fructose can be absorbed and metabolized by these cells. To identify this point, stable isotope tracing analysis was performed in SVEC4-10 cells by adding 5 mM [U-13C]-fructose to sugar-free medium containing 10% DFBS and tracing the labeled 13C in the metabolites. As shown in Fig. 2a and 2b, fructose can be metabolized to generate fructose-1-phosphate or fructose-6-phosphate, which are then metabolized in subsequent metabolic pathways. Moreover, fructose significantly increased ATP levels in VECs (Fig. 2c), and the cells produced higher levels of ATP in the medium with higher fructose concentrations (Fig. 2d). We also compared the consumption rate of fructose and glucose and the production level of lactate by VECs. For ease of detection, the glucose concentration was set to the physiological concentration of 5 mM, and the fructose concentration was also set to 5 mM. It is worth noting that the fructose consumption rate (5.8 nM/10^4^ cells/h, Fig. 2e) of VECs in fructose medium is slower than the glucose consumption rate (9.5 nM/10^4^ cells/h, Fig. 2f) in glucose medium. In the double-sugar medium, VECs also consumed fructose more slowly than glucose (glucose was 11.5 nM/10^4^ cells/h, fructose was 3.35 nM/10^4^ cells/h) (Fig. 2e and 2f), suggesting that fructose utilization is slow, but fructose accelerates glucose utilization. However, the lactate levels in the double-sugar medium were not higher than those in the glucose medium, and lactate was barely detectable in the fructose medium, suggesting that most of the consumed fructose was converted to other metabolites rather than lactate (Fig. 2g).

Meanwhile, the high-affinity fructose transporter, Glut5, and fructolysis enzymes, KHK and HK2, were all expressed in SVEC4-10 and HUVEC (Figure 2h). To further determine the effect of fructose on VEC function, HUVEC cells overexpressing Glut5 were established (Fig. 2i), and elevated Glut5 significantly increased fructose-induced proliferation (Fig. 2j), which was accompanied by increased ATP levels in the fructose medium (Fig. 2k) and a marked elevation in fructose consumption (Fig. 2l). Notably, elevated Glut5 had no significant effect on HUVEC proliferation and ATP production of cells in glucose medium (Fig. 2j and 2k). Moreover, fructose-induced proliferation of VECs was extremely suppressed in the presence of Glut5 inhibitor 2, 5-AM (2, 5-anhydro-D-mannitol) or fructolysis inhibitors (2-DG and KHK inhibitor) in fructose medium (Fig. 2m and 2n), but not in glucose medium (Fig. 2o and 2p). And the inhibitory effect of these inhibitors on VEC viability under hypoxic conditions was consistent with the previous results under normoxic conditions (Fig. S3c and S3d). Taken together, these data clearly demonstrate that fructose can be absorbed and metabolized by VECs, thereby promoting cell viability.

### Fructose enhances the migration ability and angiogenesis of VECs

**Fig. 3:**
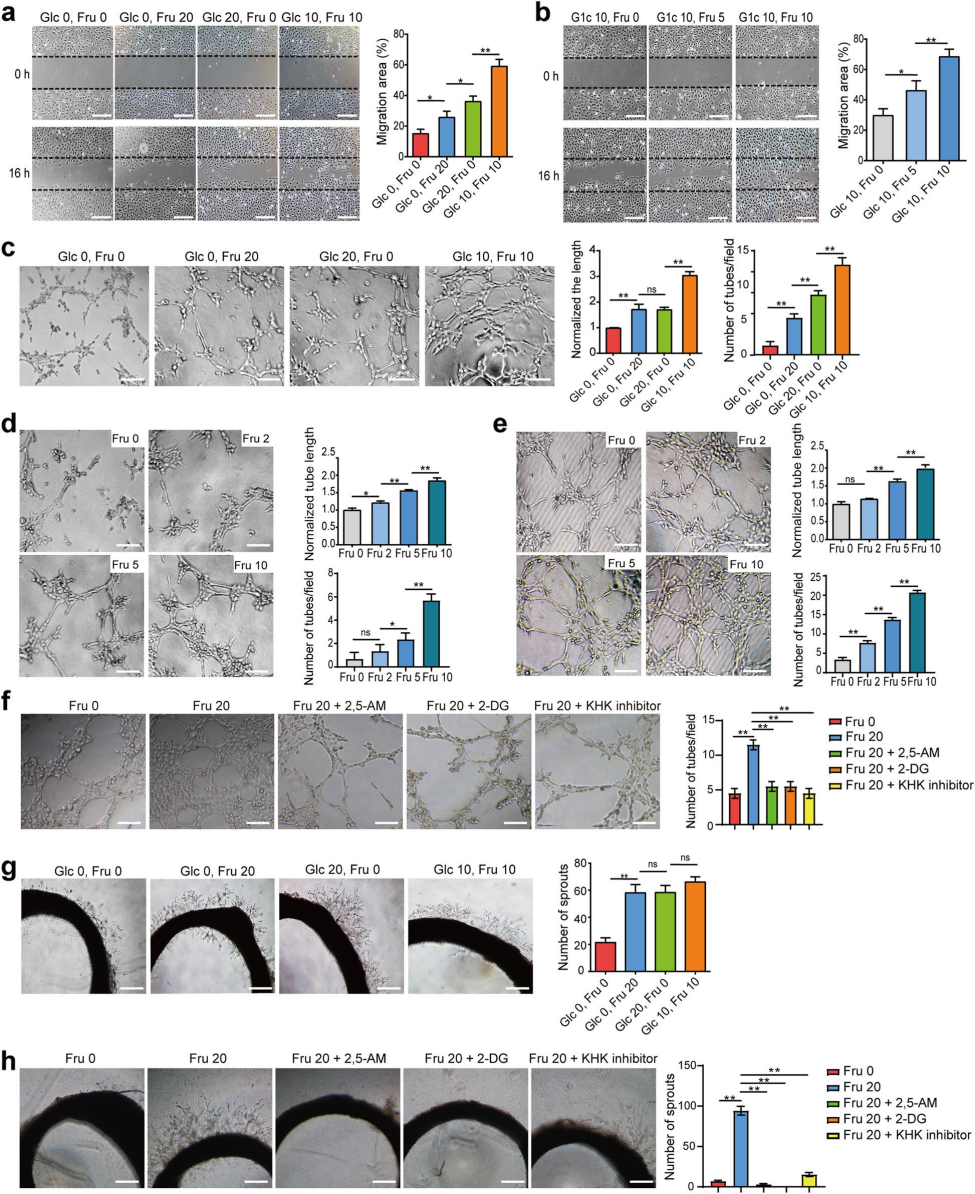
Fructose enhances the migration and angiogenesis abilities of VECs. **a**, Wound healing assay was used to analyze the migration ability of SVEC4-10 cells under the indicated culture conditions. Scale bar: 200 μm. **b**, The migration ability of SVEC4-10 cells was measured by wound healing assay in glucose (10 mM) medium containing different concentrations of fructose. Scale bar: 200 μm. **c**, Matrigel-based tube formation assay demonstrated the tube-forming ability of SVEC4-10 cells under the four specified culture conditions by measuring the length and number of tubes. Scale bar: 50 μm. **d**, The tube-forming ability of SVEC4-10 cells in glucose-free medium containing different concentrations of fructose. Scale bar: 50 μm. **e**, The tube-forming ability of SVEC4-10 cells in glucose (10 mM) medium containing different concentrations of fructose. Scale bar: 50 μm. **f**, The tube-forming ability of SVEC4-10 cells in fructose medium in the presence of 2, 5-AM (3 mM), 2-DG (2 mM) or KHK inhibitor (1 μM). Scale bar: 50 μm. **g**, Budding ability of isolated rat thoracic aorta rings under the indicated four culture conditions. Scale bar: 500 μm. **h**, The budding ability of isolated rat thoracic aortic rings in fructose medium with 2, 5-AM (3 mM), 2-DG (2 mM) or KHK inhibitor (1 μM). Scale bar: 500 μm. All data are expressed as the mean ± SD; *p < 0.05; **p < 0.01; *n *= 3

The ability of VECs to migrate is essential for angiogenesis. We examined the effect of fructose on VEC migration under four different culture conditions. The results showed that fructose promoted cell migration in sugar-free medium, and this ability was significantly enhanced in double-sugar medium (Fig. 3a and S4a). Moreover, in the presence of sufficient glucose (10 mM), cell migration was also significantly enhanced with increasing fructose concentration (Figure 3b). In addition, overexpression of Glut5 increased cell migration under fructose conditions (Fig. S4c). Interestingly, SVEC4-10 cells acquired greater migratory capacity upon prolonged exposure to fructose (Fig. S2c). We next investigated the tube-forming ability of VECs under the four different culture conditions. Compared with sugar-free medium, VECs showed a stronger tube-forming ability in fructose medium, and this ability was strongest in double-sugar medium (Fig. 3c and S4b). Moreover, the effect of fructose on tube formation of VECs was concentration-dependent in medium containing glucose (Fig. 3e) or not (Fig. 3d). In addition, the tube-forming capacity of SVEC4-10 cells exposed to fructose for a long time was significantly increased (Fig. S2d). Furthermore, Glut5 overexpression in HUVECs significantly increased the number of tubes formed in fructose medium (Fig. S4d), whereas Glut5, KHK, and HK2 inhibitors all significantly decreased tube formation (Figure 3f), indicating that fructose uptake and metabolism affected the angiogenic ability of VECs. Meanwhile, the results of the rat aortic model also revealed that fructose contributed to the growth of vascular sprouts from the aortic wall (Fig. 3g), whereas inhibition of Glut5, KHK, or HK2 distinctly hampered the growth of vascular sprouts in fructose medium (Fig. 3h). Moreover, fructose also enhanced cell migration (Fig. S5a) and tube formation (Fig. S5b) of SVEC4-10 under hypoxia, and there was no significant difference in tubulogenesis between glucose and fructose medium under hypoxic condition (Fig. S5b). Notably, the budding ability of rat aortic rings in fructose condition was higher than that in glucose medium (Fig. S5c). Therefore, all these results suggest that fructose promotes the migration and tubulogenesis of VECs under both normoxic and hypoxic conditions.

### Fructose regulates VEC activity through the AKT and Src signaling pathways

**Fig. 4:**
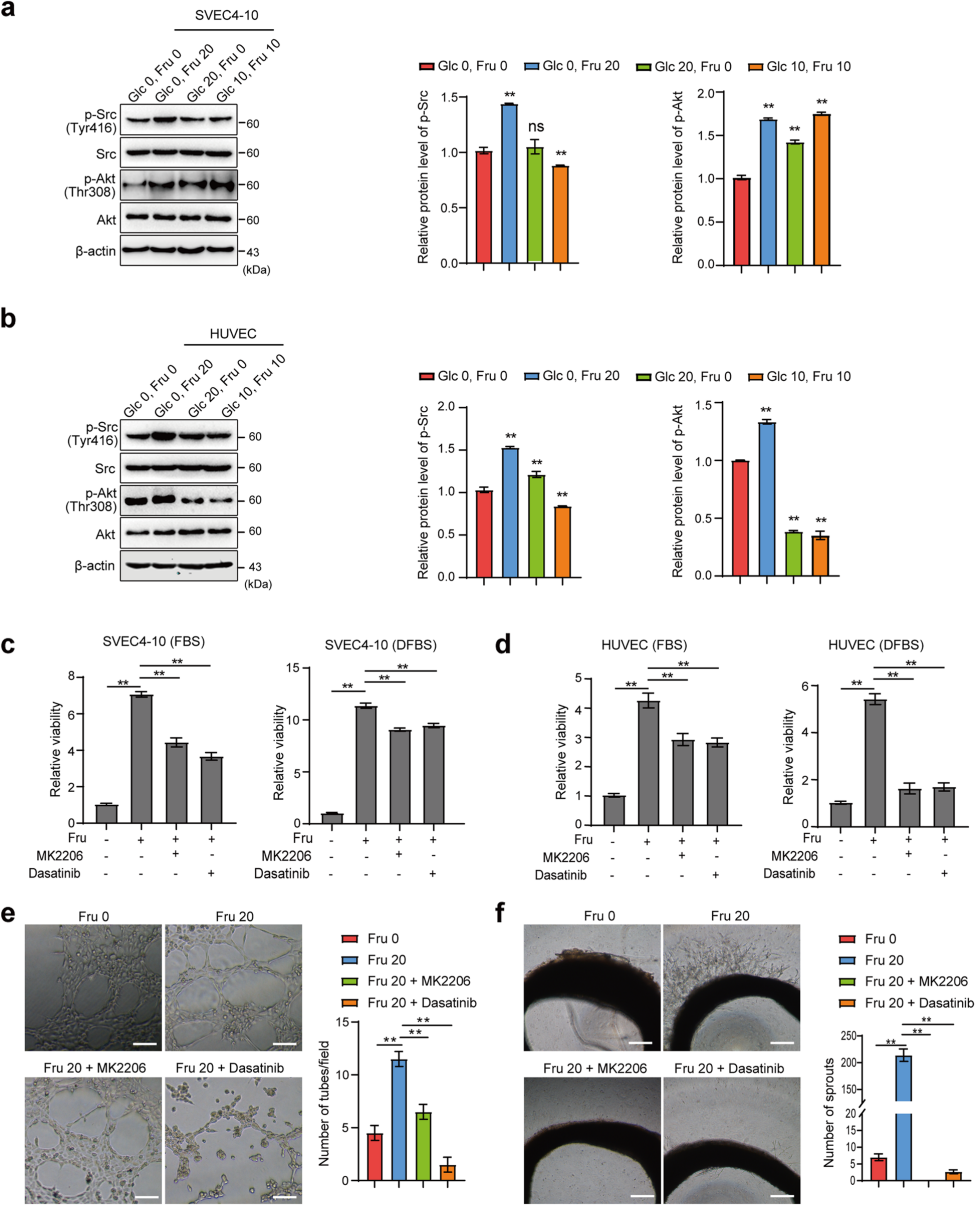
Fructose regulates the function of VECs through AKT and Src signaling pathways.** a** and** b**, Western blot analysis was performed to evaluate the activation of Akt and Src signaling pathways in SVEC4-10 (**a**) and HUVEC (**b**) cells cultured for 6 h under the indicated conditions, and the relative expression of the proteins was analyzed by grayscale using Image J. **c** and** d**, The viability of SVEC4-10 (**c**) and HUVEC (**d**) cells was analyzed by CCK-8 in fructose (20 mM) medium with the addition of the Akt pathway inhibitor MK2206 (6 μM) or the Src pathway inhibitor Dasatinib (0.3 μM) for 48 h. **e**, Tube-forming ability of SVEC4-10 cells cultured in fructose medium containing MK2206 (6 μM) or Dasatinib (0.3 μM). Scale bar: 50 μm. **f**, Budding ability of isolated rat thoracic aortic rings in fructose medium containing MK2206 (6 μM) or Dasatinib (0.3 μM). Scale bar: 500 μm. All data are expressed as the mean ± SD; ns, non-significant; *p < 0.05; **p < 0.01

To further analyze the regulatory mechanism of fructose on the activity of VECs, we examined the changes in the activation of key signaling pathways in cells under different culture conditions by Western blot analysis. The results showed that the expression levels of p-Src and p-Akt were significantly higher in the fructose group compared with the sugar-free group, and the levels of p-FAK and p-Erk were also slightly elevated, whereas the levels of p-STAT3, p-P38 and p-AMPK were not significantly changed in both SVEC4-10 (Fig. 4a and S6a) and HUVEC cells (Fig. 4b and S6b). The inhibitors MK2206 and Dasatinib, which target Akt and Src signaling, respectively (Fig. S6c and S6d), significantly inhibited cell viability (Fig. 4c and 4d) and tube-forming ability (Figure 4e), and decreased the sprouting capacity of aortic rings (Fig. 4f) in fructose medium, suggesting that the effect of fructose on angiogenesis is at least partially dependent on the activation of Akt and Src signaling pathway. In addition, the inhibitors MK2206 and Dasatinib also partially inhibited the viability of VECs in glucose medium, but the inhibition was stronger in fructose medium (Fig. S6e and S6f). In fact, our results showed that the activation of Akt and Src in VECs varied less in glucose medium than in fructose medium, so we hypothesize that glucose and fructose may have different regulatory mechanisms on the viability of VECs.

### Expression and clinical significance of Glut5 in VECs from human colorectal cancer tissues

**Fig. 5:**
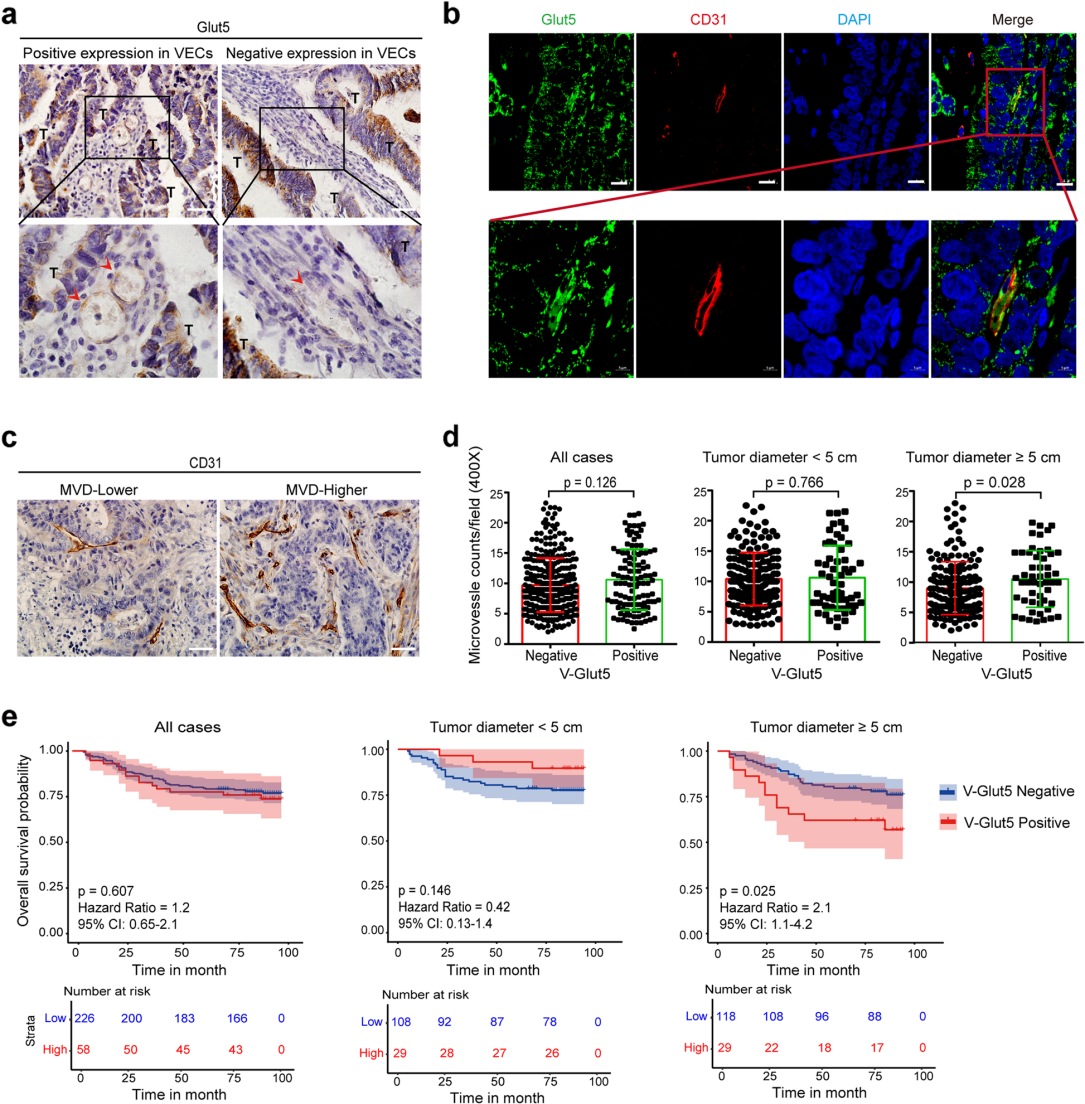
Expression and clinical significance of Glut5 in VECs of human colorectal cancer tissue.** a**, Representative images of IHC staining of Glut5 in colorectal cancer tissues, with red arrows indicating blood vessels and T indicating tumor cells. Scale bar: 50 μm. **b**, Expression and localization of Glut5 and CD31 in colorectal cancer tissues were detected by IHC staining. Scale bar: 50 μm. **c**, Representative images of CD31 staining in colorectal cancer tissues. Scale bar: 50 μm. **d**, Correlation of Glut5 expression in VECs with MVD in colorectal cancer tissues (All case: *n* = 448; Tumor diameter < 5 cm: *n* = 224; Tumor diameter ≥ 5 cm: *n* = 224). **e**, Correlation of Glut5 expression in VECs with overall survival of colorectal cancer patients (All case: *n* = 448; Tumor diameter < 5 cm: *n* = 224; Tumor diameter ≥ 5 cm: n = 224). All data are expressed as the mean ± SD; ns, non-significant; *p < 0.05; **p < 0.01

The above studies confirmed that VECs can uptake fructose through the transporter Glut5 in vitro, and we next investigated the expression of Glut5 in tumor VECs using the IHC staining method. We identified Glut5-expressing VECs in 113 out of 448 colorectal cancer tissues (Fig. 5a). Meanwhile, the results of CD31 and Glut5 immunofluorescence double staining showed that Glut5 expression was observed in CD31-positive VECs (Fig. 5b). We then analyzed the relationship between Glut5 expression in tumor VECs (V-Glut5) and MVD in cancer tissues (Fig. 5c) and found that although V-Glut5 expression did not correlate with MVD in total samples and in tumors less than 5 cm in diameter, MVD was higher in tumors with high V-Glut5 expression in tumors larger than 5 cm in diameter (Fig. 5d). Furthermore, survival analysis showed that while V-Glut5 expression was not associated with prognosis in all colorectal cancer patients and in patients with tumors less than 5 cm in diameter, patients with high V-Glut5 expression had a worse prognosis in tumors larger than 5 cm in diameter (Fig. 5e). In addition, the relationships between V-Glut5 expression and clinicopathological features in colorectal cancer patients were also analyzed, and the results showed that only age was associated with V-Glut5 expression, while there was no significant correlation between V-Glut5 expression and other features (Table 1). However, in patients with tumor diameter greater than 5 cm, V-Glut5 positive expression was significantly correlated with larger tumor size and higher clinical T-stage (Table 2). Collectively, we speculate that fructose may have a more critical role in angiogenesis in larger tumors in vivo.

**Table 1:** Correlations of V-Glut5 expression with clinicopathological features of colorectal cancer in all cases

Variables	n	V-Glut5		*χ*^2^	*p*
		Negative	Positive		
Age(years)					
60	233	165 (36.8%)	68 (15.2%)	4.534	0.038*
≥ 60	215	170 (37.9%)	45 (10.0%)		
Gender					
Male	263	199 (44.4%)	64 (14.3%)	0.15	0.740
Female	185	137 (30.6%)	48 (10.7%)		
Tumor location					
Colon cancer	224	175 (39.1%)	49 (10.9%)	2.333	0.156
Rectal cancer	224	160 (35.7%)	64 (14.3%)		
Differentiation					
well-moderate	336	249 (55.6%)	87 (19.4%)	0.571	0.529
poor	112	87 (19.4%)	25 (5.6%)		
Tumor diameter					
< 5 cm	224	167 (37.3%)	57 (12.7%)	0.048	0.913
≥ 5 cm	224	169 (37.7%)	55 (12.3%)		
T-stage					
T1-T2	112	82 (18.3%)	30 (6.7%)	0.254	0.616
T3-T4	336	253 (56.5%)	83 (18.5%)		
N-Lymphatic metastasis					
No	296	225 (49.8%)	71 (16.3%)	0.478	0.492
Yes	152	113 (25.2%)	39 (8.7%)		
M					
M0	440	330 (73.7%)	110 (24.6%)	0	1
M1	8	6 (1.3%)	2 (0.4%)		
Clinical stage					
I-II	292	219 (48.9%)	73 (16.3%)	0.210	0.649
III-IV	156	117 (26.1%)	39 (8.7%)

**Table 2:** Correlations of V-Glut5 expression with clinicopathological features of colorectal cancer in cases of tumor diameter ≥ 5 cm

Variables	n	V-Glut5		*χ*^*2*^	*p*
		Negative	Positive		
Age(years)					
< 60	122	85 (37.9%)	37 (16.5%)	4.822	0.020*
≥ 60	102	84 (37.5%)	18 (8.0%)		
Gender					
Male	139	102 (45.5%)	37 (16.5%)	0.843	0.425
Female	85	67 (29.9%)	18 (8.0%)		
Tumor location					
Colon	112	88 (39.3%)	24 (10.7%)	1.181	0.352
Rectal	112	81 (36.2%)	31 (13.8%)		
Differentiation					
well-moderate	160	122 (54.5%)	38 (17.0%)	0.195	0.731
poor	64	47 (21.0%)	17 (7.6%)		
Tumor diameter					
< 7 cm	148	118 (37.3%)	30 (12.7%)	4.320	0.038*
≥ 7 cm	76	51 (37.7%)	25 (12.3%)		
T-stage					
T1-T2	41	36 (16.1%)	5 (2.2%)	4.138	0.045*
T3-T4	183	133 (59.4%)	50 (22.3%)		
N-Lymphatic metastasis					
No	145	114 (50.9%)	31 (13.8%)	2.236	0.146
Yes	79	55 (24.6%)	24 (10.7%)		
M-Liver metastasis					
M0	220	167 (74.6%)	53 (23.7%)	1.424	0.253
M1	4	2 (0.9%)	2 (0.9%)		
Clinical stage					
I-II	144	113 (50.4%)	31 (13.8%)	1.993	0.195
III-IV	80	56 (25.0%)	24 (10.7%)		

### Effect of fructose-conditioned tumor cell medium on the biological function of VECs

**Fig. 6:**
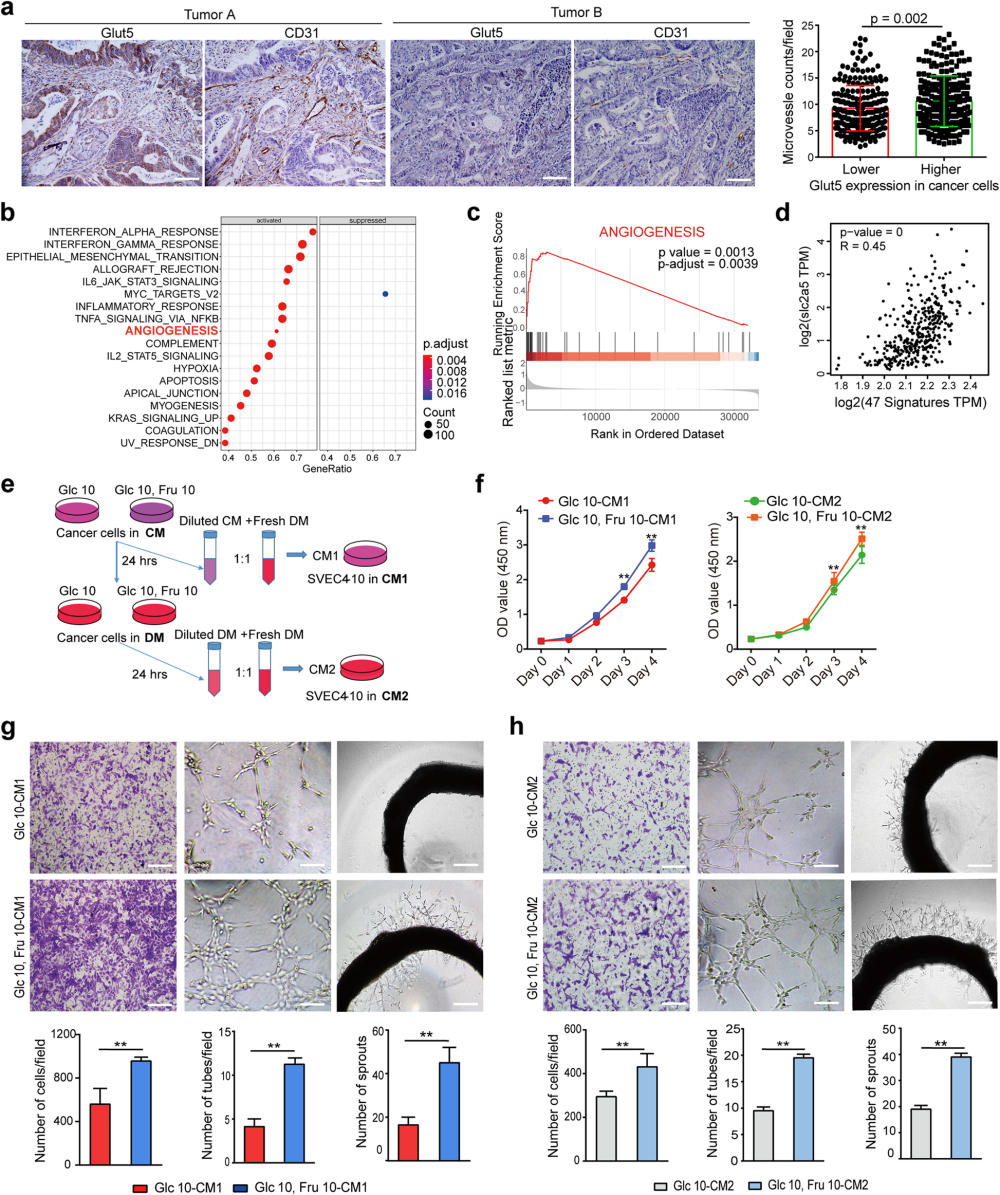
Effect of fructose-conditioned culture medium of tumor cells on the biological function of VECs. **a**, The relationship between Glut5 expression levels and MVD in colorectal cancer tissues (*n* = 448) was analyzed by IHC staining. Scale bar: 50 μm. **b**, GSEA analysis identified up- and down-regulated pathways in tissues with high Glut5 mRNA expression based on the TCGA colorectal cancer dataset. **c**, The positive correlation between angiogenesis and Glut5 mRNA. **d**, Correlation of Glut5 mRNA levels with 47 angiogenesis-related genes based on the TCGA colorectal cancer dataset, and the 47 angiogenesis-related genes were obtained in MisigDB under the HALLMARK geneset. **e**, Schematic description showing the collection of different conditional media. CM1 was fructose-containing conditional medium, and CM2 was conditional medium without fructose. **f**, Effects of the CM on the proliferation of SVEC4-10 cells. **g** and **h**, Effects of the CM1 (**g**) and CM2 (**h**) on the migration and tube-forming abilities of SVEC4-10 cells, and the budding ability of rat aortic rings. Scale bar: 100, 50 and 500 μm, respectively. All data are expressed as the mean ± SD; ns, non-significant; *p < 0.05; **p < 0.01

In analyzing the expression of Glut5 and CD31 in colorectal cancer, we found that MVD was positively correlated with the expression level of Glut5 not only in endothelial cells, but also in tumor cells (Fig. 6a). Double IHC staining of colorectal cancer specimens with antibodies against Glut5 and CD31 yielded similar results that cancer tissues with higher Glut5 expression had more abundant MVD (Fig. S7a). Meanwhile, Gene Set Enrichment Analysis (GSEA) using the TCGA colorectal cancer dataset revealed that the angiogenesis pathway was positively correlated with Glut5 mRNA expression levels (Fig. 6b and 6c). Consistently, a positive association between Glut5 expression and angiogenesis was also confirmed in the GEO colorectal cancer database (GSE32323) (Fig. S7b). In addition, there was a specific correlation between Glut5 expression levels and angiogenesis-related genes in colorectal cancer of the TCGA dataset (Fig. 6d). Indeed, Glut5 expression levels were also found to be related to angiogenesis in a variety of tumors that have been shown to be associated with fructose intake, including pancreatic cancer (Fig. S7c and S7e) and lung adenocarcinoma (Fig. S7d and S7f). These results demonstrate that fructose may indirectly regulate tumor angiogenesis by affecting the function of tumor cells, in addition to directly regulating the activity of endothelial cells.

To verify our conjecture, we cultured the colon cancer cell line CT26.WT in glucose medium (Glc 20) and double-sugar medium (Glc 10, Fru 10), respectively, and collected the two conditional medium (CM) from these cells after 24 h. These CM were then configured with normal DMEM in a 1:1 ratio to form a new medium named CM1 (i.e., Glc 20-CM1 and Glc 10, Fru 10-CM1). Afterward, the above cells were replaced with fresh DMEM and further cultured for 24 h. These culture supernatants were then collected and configured with fresh DMEM in a 1:1 ratio to form another new medium named CM2 (i.e., Glc 20-CM2 and Glc 10, Fru 10-CM2) (Fig. 6e). Subsequently, these CMs were applied to culture SVEC4-10 and the rat aortic rings, and the results showed that, compared with Glc 20-CM, both Glc 10, Fru 10-CM1 and Glc 10, Fru 10-CM2 significantly promoted the proliferation (Fig. 6f), migration and tube formation of SVEC4-10 cells (Fig. 6g and 6h), and increased the outgrowth capacity of the aortic ring (Fig. 6g and 6h). Taken together, these results suggest that tumor cells grown in fructose-containing medium have a greater ability to accelerate angiogenesis than those grown in glucose medium alone.

### Fructose promotes VEGF expression by affecting ROS and HIF1α in tumor cells

**Fig. 7:**
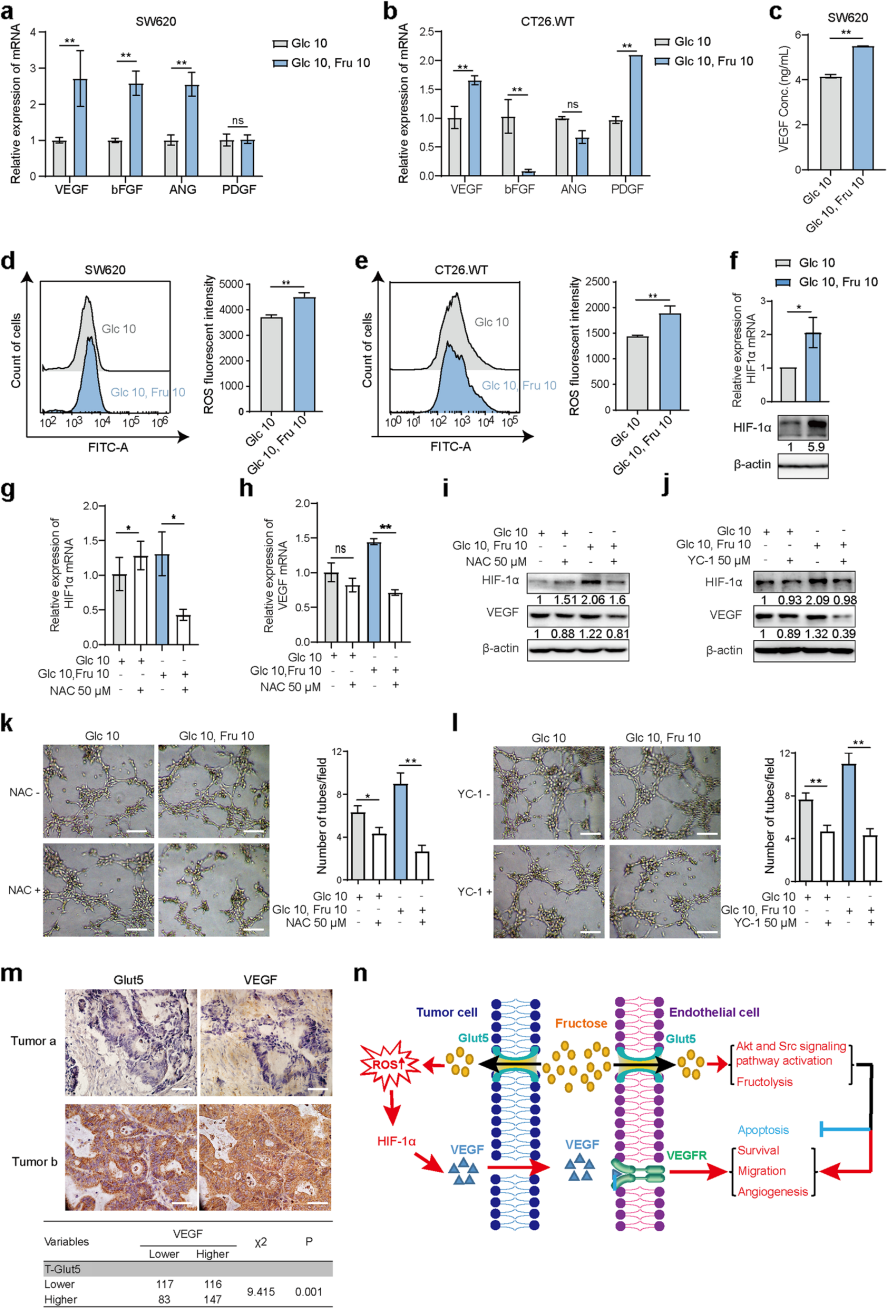
Fructose promotes VEGF expression by affecting ROS and HIF1α in tumor cells.** a** and **b**, Intracellular mRNA levels of VEGF, bFGF, ANG and PDGF were detected by qRT-PCR in SW620 (**a**) and CT26.WT (**b**) cells cultured in media for 24 h. **c**, VEGF levels in the supernatants of the above SW620 media detected by ELISA. **d** and **e**, ROS levels in SW620 (**d**) and CT26.WT cells (**e**) cultured in media for 24 h. **f**, The expression level of HIF1α in SW620 cells cultured in media for 24 h. **g** and **h**, Effect of NAC (50 μM) on HIF1α (**g**) and VEGF (**h**) mRNA expression in SW620 cells cultured in media for 24 h. **i**, Effect of NAC (50 μM) on HIF1α and VEGF protein expression in SW620 cells cultured in the above two media for 24 h. **j**, Effect of YC-1 (50 μM) on HIF1α and VEGF expression in SW620 cells cultured in media for 24 h. Grayscale was normalized to the mean of the no inhibitor and glucose-free groups. **k** and **l**, SW620 cells were cultured in two media (Glc 10 mM; Glc 10 mM, Fru 10 mM) in the presence of NAC (**k**) or YC-1 (**l**) for 24 h, respectively, and then the medium was replaced with normal medium for 12 h. These media were collected as CM for tube formation assays. Scale bar: 50 μm. **m**, IHC staining was used to detect the expression of Glut5 and VEGF in tumor cells of human colorectal cancer tissues, and the relationship between the expression levels of Glut5 and VEGF was statistically analyzed (*n* = 463). Scale bar: 20 μm. **n**, Schematic diagram of the molecular mechanism proposed in this study. All data are expressed as the mean ± SD; ns, non-significant; *p < 0.05; **p < 0.01

To explore the mechanism by which fructose-cultured tumor cells regulate angiogenesis, we determined the levels of various pro-angiogenic factors, such as VEGF, bFGF, ANG and PDGF, in tumor cells under different culture conditions. The results of real-time PCR showed that the expression level of VEGF mRNA in SW620 and CT26.WT cell lines was significantly higher in double-sugar medium than in the glucose medium (Fig. 7a and 7b). Subsequent ELISA tests showed the similar results (Fig. 7c), suggesting that fructose could upregulate VEGF expression in colon cancer cells. Hypoxia-inducible factor 1-alpha (HIF1α) is known to be a key regulator of VEGF expression by directly binding to the *VEGF* promoter [29]. Multiple studies have shown that fructose can produce large amounts of reactive oxygen species (ROS) during the metabolic processes of various cells [30, 31], and ROS has also been reported to upregulate HIF1α expression [32, 33]. Therefore, we further investigated whether fructose-induced upregulation of VEGF expression was related to ROS and HIF1α in colon cancer cells. As shown in Fig. 7d and 7e, the ROS levels of SW620 and CT26.WT in double-sugar medium were significantly higher than those in glucose medium, and the cells in the double-sugar medium expressed higher levels of HIF1α (Fig. 7f). Notably, ROS removal by N-acetylcysteine (NAC) significantly inhibited the mRNA expression of HIF1α and VEGF in SW620 cells in double-sugar medium (Figure 7g and 7h), and the protein levels of both molecules were also downregulated (Fig. 7i). Meanwhile, the HIF1α inhibitor YC-1 could also significantly downregulate the expression of VEGF in cells under double-sugar medium (Fig. 7j). Therefore, these results demonstrate that the upregulation of VEGF expression by fructose is related to the higher level of ROS-induced upregulation of HIF1α. CMs collected from SW620 cells pretreated with NAC or YC-1 in fructose medium failed to promote the tube-forming ability of HUVECs (Fig. 7k and 7l). In addition, to further verify the regulatory effect of fructose metabolism on VEGF expression in tumor cells, we detected the relationship between Glut5 and VEGF expression in colorectal cancer tissues by IHC staining, and found that high expression of Glut5 was accompanied by high expression of VEGF in tumor cells (Fig. 7m). Collectively, our results demonstrate that fructose promotes VEGF expression by affecting ROS and HIF1α in tumor cells, which in turn contributes to tumor angiogenesis.

## Discussion

Metabolic reprogramming of tumors enables them to utilize as much of all available nutrients as possible to generate energy and biomacromolecules required for rapid proliferation [34]. This metabolic plasticity occurs not only in tumor cells, but also in stromal cells in the tumor microenvironment, such as VECs [23, 35–37]. Fructose is a very common sugar found in natural and processed foods and is an alternative carbon source that some cells can use for glycolysis. Although significant associations between high fructose intake and increased risk of several cancers and more aggressive tumor behavior have been widely reported [2, 3, 11, 38, 39], the mechanisms involved remain elusive. In this study, we demonstrate that fructose can enhance the angiogenic capacity of VECs by maintaining cell survival and promoting their migration and tube-forming ability. We provide evidence that the fructolytic proteins Glut5, KHK, and HK2 are all expressed in two endothelial cell lines, and that fructose can be absorbed and metabolized by these cells and contribute to tumor angiogenesis. Additionally, we also confirmed the correlation of Glut5 expression in VECs with angiogenesis in colorectal cancer specimens. Moreover, fructose can also indirectly enhance the function of VECs by upregulating the expression of VEGF in colorectal cancer cells. This pro-VEGF secretion effect of fructose is achieved by affecting the level of ROS and the expression of HIF1α in tumor cells. These results were further validated in several solid tumor tissues including colorectal, pancreatic and lung cancer, and in mouse models. Collectively, our study demonstrates that fructose promotes tumor progression by affecting angiogenesis. Our findings also suggest that targeting fructose metabolism may be a potential tumor therapy strategy.

Our findings support the role of fructose in accelerating tumor progression by promoting angiogenesis. Although recent studies have confirmed that increased fructose intake promotes the occurrence and progression of multiple tumors, most studies have focused on the utilization and metabolism of fructose by cancer cells [4, 7, 12, 16, 40], while the relevance of fructose to angiogenesis in the microenvironment has received little attention. In this study, we found in three xenograft models that in addition to promoting tumor growth in fructose-fed mice, there was a significant increase in MVD in tumor tissue, suggesting an association between fructose and angiogenesis. This possibility is partly supported by a recent study reporting that increased fructose intake was positively correlated with the angiogenic biomarker sVCAM-1 in cancer patients [41]. Moreover, fructose also maintained VEC survival and promoted their migratory and tube-forming abilities in the absence of glucose, which are essential for VECs to generate blood vessels. Although fructose had less effect on VEC function than glucose under normoxic conditions, it still improved VEC viability under glucose-sufficient conditions.

Notably, we found that fructose had a more pronounced effect on regulating tubulogenesis and aortic ring sprouting than glucose under hypoxic conditions. Given that the tumor microenvironment in vivo is often hypoxic and that hypoxia can directly induce angiogenesis [42], fructose may play a more critical role in tumor angiogenesis in vivo. Indeed, our xenograft studies showed that either glucose or fructose accelerated tumor growth, while only fructose significantly promoted tumor angiogenesis. Therefore, these results suggest that fructose promotes angiogenesis by directly enhancing the function of VECs. Higher vascular density provides more nutrients and oxygen to promote tumor growth [43]. Consistently, the Ki67 index was higher in the tumor tissue of mice fed with fructose water, and the MVD was positively correlated with the Ki67 index. In addition, we also found that expression of the fructose-specific transporter Glut5 was positively expressed in VECs of human colorectal cancer tissues, and that elevated V-Glut5 expression was positively associated with higher MVD and poorer prognosis, particularly in larger tumors. These data suggest that fructose may play a more important role in tumor progression, possibly by promoting angiogenesis to accelerate tumor progression. Taken together, our data suggest a strong association between high fructose consumption and tumor angiogenesis, which is another important piece of evidence that fructose promotes tumor progression.

Although previous studies have shown that cancer cells can uptake and metabolize fructose [10, 11, 16], it remains to be determined whether VECs can utilize fructose. In the present study, the metabolic flux analysis of ^13^C-labeled fructose confirmed that VECs are able to take up and metabolize fructose. Fructose also increased ATP levels in VECs in a time- and concentration-dependent manner, and regulated the activation of key signaling pathways. Thus, these data demonstrate that fructose is a nutrient that can be utilized by VECs. Subsequent studies showed that VECs do express certain levels of key molecules capable of transporting and metabolizing fructose, which further supports the idea that VECs can utilize fructose. However, whether fructose metabolism by VECs affects angiogenesis remains to be clarified. This speculation was supported by subsequent intervention studies in which inhibition of fructose transport with inhibitors of Glut5, as well as inhibition of fructose metabolism with inhibitors of KHK and HK2, significantly inhibited endothelial cell viability under fructose-rich conditions. Moreover, the ability of VECs to migrate and form tubes, as well as the ability of rat arterial rings to sprout, were reduced in the presence of these inhibitors. It is noteworthy that VECs utilize fructose less efficiently than glucose in both single-sugar and double-sugar media under normal culture conditions. We speculate that VECs preferentially utilize glucose in the presence of sufficient nutrients, but still retain the ability to metabolize fructose to ensure that cell viability is maintained in the absence of glucose. Interestingly, this hypothesis was supported by our subsequent analysis, in which fructose utilization by VECs was markedly enhanced after prolonged glucose deprivation, along with an increase in angiogenic capacity. This mechanism of fructose metabolism in endothelial cells may become more important in the context of intra-tumor nutritional deficiency. It is likely that in smaller tumors the nutrient supply is relatively adequate. Angiogenesis may not be very active, and the effect of fructose is not obvious. Conversely, the role of fructose in tumor angiogenesis becomes critical when the tumor is large and nutrient deprivation is excessive and relatively inadequate. This concept was supported by the analysis of clinical tumor specimens, in which the expression of fructose-specific transporter V-Glut5 was positively correlated with higher MVD in larger tumors. Collectively, these results suggest that the ability of endothelial cells to metabolize fructose is necessary for angiogenesis, especially in the presence of glucose deficiency.

Another important finding of our study is that tumor cells utilizing fructose can indirectly promote angiogenesis. Although fructose is known to be metabolized by cancer cells, the effect of this metabolism on VECs in cancer cells has not been explored. Here, we found that MVD was more abundant in tissues with higher Glut5 expression in the tumor cells of colorectal cancer specimens. Similarly, GSEA analysis showed that Glut5 expression was positively correlated not only with the angiogenic pathway in a variety of tumors, but also with 47 angiogenesis-related gene signature. These findings suggest an association between fructose utilization in cancer cells and angiogenesis. In agreement, conditioned medium from tumor cells cultured in fructose-containing medium has a greater ability to promote tumor angiogenesis than glucose medium alone. Tumor cells are known to be able to secrete various pro-angiogenic factors (e.g., VEGF) to induce angiogenesis [44, 45]. Interestingly, fructose can upregulate the expression of VEGF in colon cancer cells, and that the expression of Glut5 and VEGF were positively correlated in colorectal cancer tissues, suggesting that the increase in fructose-induced angiogenesis may be related to its promotion of VEGF expression. Fructose can generate large amounts of ROS during metabolism [30, 31, 46], and the ROS-HIF1α pathway is known to upregulate VEGF expression in various cells [32]. As expected, compared with cells cultured with glucose alone, fructose-containing medium significantly increased the level of intracellular ROS, as well as increased the expression of HIF1α. These data suggest that fructose may upregulate VEGF expression in cancer cells through the ROS-HIF1α signaling pathway. In support of this hypothesis, quenching of ROS with NAC and inhibition of HIF-1α with YC-1 both significantly reduced fructose-induced upregulation of VEGF expression. Moreover, the addition of NAC and YC-1 to the medium also significantly inhibited the promoting effect of fructose on the tube-forming ability of HUVECs. Collectively, our results demonstrate that fructose can promote VEGF expression by regulating the ROS-HIF1α signaling pathway in tumor cells, thereby indirectly inducing angiogenesis.

## Conclusions

In conclusion, as shown in Fig. 7n, we found that fructose not only enhances the biological function of VECs by providing nutrients and activating key signaling pathways, but also upregulates the expression of VEGF in tumor cells by increasing ROS, which ultimately promotes colorectal cancer angiogenesis. Therefore, we believe that if the metabolism of VECs is the engine of angiogenesis, then the fructose metabolic reprogramming of VECs and tumor cells will add horsepower to this engine. In general, fructose metabolism plays multiple roles in tumors, and targeting fructose metabolism may be a potential cancer treatment strategy to both attack tumor cells and inhibit tumor angiogenesis.

## Materials and methods

### Mouse models

Four-week-old male BALB/c mice were purchased from Jiangsu Collective Pharmachem Biotechnology Co. and inoculated subcutaneously with CT26.WT or MC38 cells. After one week, the mice were randomly divided into three groups (*n* = 5 per group) and fed with distilled water, 10% glucose water, or 10% fructose water, respectively. Tumor volumes were recorded starting one week after inoculation. Tumor length and width were measured in vitro every three days using vernier calipers. Three weeks after inoculation, mice were euthanized with carbon dioxide and tumors were dissected, rinsed, imaged and fixed. H&E staining and immunohistochemical staining were then performed to detect the expression of CD31, VEGF and Ki67 in the tumor sections. All experimental operations were performed in accordance with the requirements of the Animal Ethics Committee of the Institute of Oncology of Tianjin Medical University Cancer Institute and Hospital.

### Tube formation assay

A 96-well plate was coated with matrigel (BD Biosciences, San Jose, CA) (50 μL per well) and incubated at 37 °C incubator for 30 min. Then VECs (3.0 × 10^4^ cells per well) in 200 μL conditioned medium were added to the gel for 4 h at 37 °C. The formation of capillary-like structures in each well was evaluated and photographed using a microscope. For quantification, the total length of tubes and the number of tubes were calculated and statistically analyzed.

### Rat aorta model of angiogenesis

Six-week-old Sprague Dawley rats were selected and the aorta was removed after ether anesthesia. The periaortic fat was separated by immersion in sterile PBS, and the aorta was cut into 1–1.5 mm thick sections and placed in a 96-well plate. Matrigel diluted 1:1 with the corresponding medium was added into each well at 70 μL, and the plate was coagulated at 37 °C for 1 h. Then 200 μL of conditioned medium was added to the gel, and the 96-well plate was incubated at 37 °C in an incubator for 3–5 days. Microvascular outgrowth was detected by a microscopy, and the number of sprouts was calculated.

### Isotope tracing analysis with 13C-labeled fructose

SVEC4-10 cells were incubated in 10 cm medium for wall attachment for 24 h, then washed with PBS and replaced with glucose-free medium supplemented with 10% dialysis FBS, 10 mM [U-13C6] fructose and 2 mM glutamine for 8 h. To harvest intracellular metabolites, the medium was aspirated and the metabolism quenched with 80% cold methanol, and the resulting lysate was stored at -80 °C for 2 h. The cell lysats was removed by centrifugation at 12000 g at 4 °C for 20 min to remove macromolecules and debris, and then the supernatant was placed in a nitrogen stream until it dried into powder by using the characteristics of low temperature drying in nitrogen. The dried samples can be stored at -80 °C. The samples were sent to the Experimental Technology Center of the Protein Facility of Tsinghua University for LC/MS analysis. Briefly, metabolites were resuspended in 50 μL of 80% methanol and 1 μL of each sample was injected onto a Synergi Hydro-RP 100A 2.1 × 100 mm column (Phenomenex, Torrance, CA, USA) at 35 °C Metabolite separation was performed at the column temperature. Multiple reaction monitoring data were analyzed using Tracefinder (Thermo Fisher Scientific) to quantify metabolites for throughput analysis. Finally, the relative abundance of metabolites was compared between samples.

### Fructose, Glucose and lactate concentration detection

The M-100 Automatic Biosensors Analyzer (Shenzhen Siemen Technology Co., Ltd.) was used to detect the concentrations of lactate and glucose in cell culture medium according to manufacturer's instructions. The concentration of fructose in the medium was detected using the Fructose Assay Kit (BioVision, #K619-100) according to the instructions. Briefly, 1.5 × 10^5^ cells per well were seeded into 12-well plates, and the corresponding medium was added after cell attachment. The cell media were collected at 0 h and 8 h, and the concentrations of fructose, glucose, and lactate were measured. The sugar consumption and lactate production were compared between groups according to metabolic time and cell number.

### Patients and clinical samples

A total of 448 colorectal cancer tissues were collected from patients diagnosed at Tianjin Medical University Cancer Institute and Hospital between 2011 and 2013. All retrospective clinicopathological information of patients were collected, including sex, age, tumor size, lymph node metastasis, TNM stage, and pathological differentiation. Survival analysis was calculated using Kaplan–Meier analysis and log-rank test. The use of these specimens and patient information was approved by the Ethics Committee of Tianjin Medical University Cancer Institute and Hospital.

### Statistical analysis

Analysis of differences between data groups was performed using the Unpaired Student's t-test, one-way or two-way ANOVA test in GraphPad Prism 9.00 software. Correlations of Glut5 expression with patient’s prognosis and clinicopathological parameters were analyzed using the IBM SPSS 13.0 software. *P* < 0.05 was considered statistically significant. Each experiment was performed in triplicate, and all data are expressed as the mean ± standard deviation. Other methods are available in the supplemental dataset.

## Supplementary Information

**Additional file 1. **Supplementary materials and Methods

**Additional file 2: Fig. S1. **Fructose promotes tumor angiogenesis* in vivo* and VEC viability *in vitro. ***a**, The volume of MC38 subcutaneous tumors in the indicated groups of mice (5 mice per group). **b**, Representative images of CD31 IHC staining of dissected tumors. Scale bar: 20 μm. **c**, MVDs counted by CD31 staining in dissected tumors. Scale bar: 20 μm. **d**, Correlation analysis between MVD and tumor volume. **e**, Ki67 positive index in tumor tissues of each group was detected by IHC staining. Scale bar: 20 μm. **f**, The percentages of Ki67-positive cells/all cancer cells were calculated. g, Correlation analysis between MVD and Ki‑67 proliferation index in tumors. h, Trypan blue staining was used to count the number of viable cells in different media to assess cell proliferation.

**Additional file 3: Fig. S2. **Changes in the biological behaviors of VECs cultured in fructose medium for 2 weeks. a and b, The proliferation ability of SVEC4-10 cells in different media (Glc 0, Fru 20: 20 mM fructose only; Glc 20, Fru 0: 20 mM glucose only; Glc 10, Fru 10: 10 mM each of glucose and fructose) containing 10% DFBS (a) or FBS (b) after long-term fructose induction. c, The migration ability of fructose-induced SVEC4-10 cells in the different media. Scale bar: 100 μm. d, The tube-forming ability of fructose-induced SVEC4-10 cells in the different media. Scale bar: 50 μm. All data are expressed as the mean ± SD; ns, non-significant; *p < 0.05; **p < 0.01; *n* = 3.

**Additional file 4: Fig. S3.** Effects of fructose on VEC viability under hypoxic condition. a, CCK-8 analysis of the viability of SVEC4-10 cells cultured in glucose-free medium containing different concentrations of fructose, supplemented with 10% FBS or DFBS. b, Cell death analysis of SVEC4-10 cells cultured for 24 h in glucose-free medium with or without fructose (20mM). c, CCK-8 analysis of the viability of SVEC4-10 and HUVEC cells cultured in 20 mM fructose-containing medium in the presence of 2, 5-AM (3 mM), 2-DG (2 mM) or KHK inhibitor (1 μM) for 24 h under hypoxic condition. d, CCK-8 analysis of the viability of SVEC4-10 and HUVEC cells cultured in 20 mM glucose-containing medium in the presence of 2, 5-AM (3 mM), 2-DG (2 mM) or KHK inhibitor (1 μM) for 24 h under hypoxic condition. All data are expressed as the mean ± SD; ns, non-significant; *p < 0.05; **p < 0.01. n = 3.

**Additional file 5: Fig. S4. **Fructose promotes the migration and angiogenesis of HUVEC cells. a, The migration ability of HUVEC cells in four types of media (Glc 0, Fru 0: no glucose and fructose; Glc 0, Fru 20: 20 mM fructose only; Glc 20, Fru 0: 20 mM glucose only; Glc 10, Fru 10: 10 mM each of glucose and fructose) was analyzed by wound healing assay. Scale bar: 200 μm. b, Tube formation assay was used to analyze the angiogenic ability of HUVEC cells in four types of media. Scale bar: 50 μm. c and d, The migration (c) and tube-forming (d) abilities of HUVEC cells overexpressing Glut5 were assayed under different culture conditions. Scale bar: 200 μm (c) and 50 μm (d). All data are expressed as the mean ± SD; ns, non-significant; *p < 0.05; **p < 0.01; n = 3.

**Additional file 6: Fig. S5.** Effects of fructose on biological functions of VECs under hypoxia. a, Wound healing assay was performed to analyze the migration ability of SVEC4-10 cells cultured in four different media under hypoxia for 16 h. Scale bar: 200 μm. b, The tube-forming ability of SVEC4-10 cells was analyzed under the indicated culture conditions under hypoxia for 4 h. Scale bar: 50 μm. c, The budding ability of rat aortic rings under the four culture conditions under hypoxia for 4 days. Scale bar: 500 μm. All data are expressed as the mean ± SD; ns, non-significant; *p < 0.05; **p < 0.01; n = 3.

**Additional file 7: Fig. S6. **Effect of fructose on key signaling pathways in VECs. a and b, SVEC4-10 (a) and HUVEC (b) cells were cultured in four types of media for 2 h, 6 h and 12 h, and then the activation of signaling pathways was detected by Western blot. Gray analysis was performed using Image J software, and the gray scale of each band was normalized to the mean value of that in the Glc 0, Fru 0 (2 h) group. c and d, Effects of different concentrations of MK2206 and Dasatinib on the activities of AKT and Src signaling pathways after treatment of cells for 2 h. Gray analysis was performed using Image J software, and the gray scale was normalized to the mean value of that in the no inhibitor group. e and f, CCK-8 assay was used to analyze the viability of SVEC4-10 (e) and HUVEC (f) cells cultured in 20 mM glucose medium with MK2206 (6 μM) or Dasatinib (0.3 μM) for 48 h. All data are expressed as the mean ± SD; ns, non-significant; *p < 0.05; **p < 0.01. n = 3.

**Additional file 8: Fig. S7.** Relationship between Glut5 mRNA expression levels and angiogenesis in tumor tissues. a, Representative images of double IHC staining of colorectal cancer tissues using Glut5 and CD31 antibodies. Glut5-positive cancer cells are stained red and CD31-stained microvasculars are stained brown, with red arrows indicating blood vessels. Scale bar: 50 μm. b, GSEA analysis identified up- and down-regulated pathways in tissues with high Glut5 mRNA expression based on the colorectal cancer dataset GSE32323. c and d, GSEA analysis identified up-regulated pathways in tissues with high Glut5 mRNA expression based on the TCGA dataset of pancreatic cancer (c) and lung adenocarcinoma (d). e and f, Correlation of 47 angiogenesis-related genes with Glut5 mRNA expression based on the TCGA dataset of pancreatic cancer (e) and lung adenocarcinoma (f). Analysis was performed using a two-tailed Pearson correlation analysis (e,f)

## Data Availability

Data are available on reasonable request. All data relevant to the study are included in the article or uploaded as online supplemental information.

## Abbreviations

Akt: Protein Kinase B, PKB
AMPK: Adenosine 5 ‘-monophosphate (AMP)-activated protein kinase
ANG: Angiopoietin
ATP: Adenosine 5'-triphosphate
bFGF: Basic fibroblast growth factor
CCK-8: Cell Counting Kit-8
CD31: Platelet endothelial cell adhesion molecule-1,PECAM-1/CD31
CM: Conditional medium
DFBS: Dialyzed Fetal Bovine Serum
DMEM: Dulbecco's modified eagle medium
EdU: 5-Ethynyl-2’-deoxyuridine
ELISA: Enzyme-Linked Immunosorbent Assay
Erk: Extracellular regulated protein kinases
FAK: Focal Adhesion Kinase
FBS: Fetal Bovine Serum
GEO: Gene Expression Omnibu
Glut5: Glucose transporter 5
GSEA: Gene Set Enrichment Analysis
H&E: Hematoxylin–eosin staining
HFCS: High fructose corn syrup
HIF1α: Hypoxia-inducible factor 1-alpha
HK2: Hexokinase 2
IHC: Immunohistochemistry
KHK: Ketohexokinase
LC/MS: Liquid chromatography tandem mass spectrometry
mRNA: Messenger Ribonucleic Acid
mTORC1: Mammalian target of rapamycin complex 1
MVD: Microvascular density
NAC: N-Acetylcysteine
PCR: Polymerase chain reaction
PDGF: Platelet-derived growth factor
PI: Propidium Iodide
P38: P38 mitogen-activated protein kinases
ROS: Reactive oxygen species
SPSS: Statistical Product and Service Solutions
Src: Proto-oncogene, non-receptor tyrosine kinase
sVCAM-1: Soluble vascular cell adhesion molecule-1
STAT3: Signal transducers and activators of transcription 3
TCGA: The Cancer Genome Atlas
TNM: Tumor Node Metastasis
VECs: Vascular endothelial cells
VEGF: Vascular endothelial growth factor
YC-1: Lificiguat
2, 5-AM: 2, 5-Anhydro-D-mannitol
2-DG: 2-Deoxy-D-glucose

## References

[1] Johnson RJ (2020). Fructose metabolism as a common evolutionary pathway of survival associated with climate change, food shortage and droughts. J Intern Med.

[2] Santhekadur PK (2020). The dark face of fructose as a tumor promoter. Genes Dis.

[3] Krause, N. and A. Wegner, Fructose Metabolism in Cancer. Cells, 2020. 9(12). 10.3390/cells9122635 PMC7762580 33302403

[4] Jiang H (2021). Fructose and fructose kinase in cancer and other pathologies. J Genet Genomics.

[5] Zhao S (2020). Dietary fructose feeds hepatic lipogenesis via microbiota-derived acetate. Nature.

[6] Jang C (2020). The small intestine shields the liver from fructose-induced steatosis. Nat Metab.

[7] Goncalves MD (2019). High-fructose corn syrup enhances intestinal tumor growth in mice. Science.

[8] Febbraio MA, Karin M (2021). "Sweet death": Fructose as a metabolic toxin that targets the gut-liver axis. Cell Metab.

[9] Jang C (2018). The small intestine converts dietary fructose into glucose and organic acids. Cell Metab..

[10] Liu H (2010). Fructose induces transketolase flux to promote pancreatic cancer growth. Cancer Res.

[11] Nakagawa T (2020). Fructose contributes to the Warburg effect for cancer growth. Cancer Metab.

[12] Shen Z (2022). GLUT5-KHK axis-mediated fructose metabolism drives proliferation and chemotherapy resistance of colorectal cancer. Cancer Lett.

[13] Chica-Cid, T. and M.D. Giraldez, Untangling the Contribution of Fructose Metabolism to Obesity and Colorectal Cancer: A Tale of Adaptation to Hypoxia in the Gut. Gastroenterology, 2022. 10.1053/j.gastro.2022.06.022 35697138

[14] Weng Y (2018). SLC2A5 promotes lung adenocarcinoma cell growth and metastasis by enhancing fructose utilization. Cell Death Discov.

[15] Chen, W.L., et al., GLUT5-mediated fructose utilization drives lung cancer growth by stimulating fatty acid synthesis and AMPK/mTORC1 signaling. JCI Insight, 2020. 5(3). 10.1172/jci.insight.131596 PMC7098789 32051337

[16] Jeong S (2021). High Fructose Drives the Serine Synthesis Pathway in Acute Myeloid Leukemic Cells. Cell Metab..

[17] Monzavi-Karbassi B (2010). Fructose as a carbon source induces an aggressive phenotype in MDA-MB-468 breast tumor cells. Int J Oncol.

[18] Kargozar S (2020). Nanotechnology for angiogenesis: opportunities and challenges. Chem Soc Rev.

[19] De Palma M, Biziato D, Petrova TV (2017). Microenvironmental regulation of tumour angiogenesis. Nat Rev Cancer.

[20] Du, W., et al., Endothelial cell glucose metabolism and angiogenesis. Biomedicines, 2021. 9(2). 10.3390/biomedicines9020147 PMC7913320 33546224

[21] Abumrad NA (2021). Endothelial cell receptors in tissue lipid uptake and metabolism. Circ Res.

[22] Eelen G (2018). Endothelial cell metabolism. Physiol Rev.

[23] Li X, Sun X, Carmeliet P (2019). Hallmarks of endothelial cell metabolism in health and disease. Cell Metab.

[24] Schaaf MB (2019). Autophagy in endothelial cells and tumor angiogenesis. Cell Death Differ.

[25] Bruning U (2018). Impairment of Angiogenesis by Fatty Acid Synthase Inhibition Involves mTOR Malonylation. Cell Metab.

[26] Joyal JS (2016). Retinal lipid and glucose metabolism dictates angiogenesis through the lipid sensor Ffar1. Nat Med.

[27] Akbarian M, Bertassoni LE, Tayebi L (2022). Biological aspects in controlling angiogenesis: current progress. Cell Mol Life Sci.

[28] Cho HY (2021). Energy and sugar signaling during hypoxia. New Phytol.

[29] Bhattarai D, Xu X, Lee K (2018). Hypoxia-inducible factor-1 (HIF-1) inhibitors from the last decade (2007 to 2016): A "structure-activity relationship" perspective. Med Res Rev.

[30] Lu XL (2017). Quercetin attenuates high fructose feeding-induced atherosclerosis by suppressing inflammation and apoptosis via ROS-regulated PI3K/AKT signaling pathway. Biomed Pharmacother.

[31] Jaiswal N (2015). Fructose-induced ROS generation impairs glucose utilization in L6 skeletal muscle cells. Free Radic Res.

[32] Vanderstraeten J (2018). Acute iodine deficiency induces a transient VEGF-dependent microvascular response in mammary glands involving HIF-1, ROS, and mTOR. Am J Physiol Cell Physiol.

[33] Zhang J (2021). Retraction Note to: chrysophanol exhibits anti-cancer activities in lung cancer cell through regulating ROS/HIF-1a/VEGF signaling pathway. Naunyn Schmiedebergs Arch Pharmacol.

[34] Martinez-Reyes I, Chandel NS (2021). Cancer metabolism: looking forward. Nat Rev Cancer.

[35] Peng H (2021). Metabolic reprogramming of vascular endothelial cells: basic research and clinical applications. Front Cell Dev Biol.

[36] Li X, Kumar A, Carmeliet P (2019). Metabolic pathways fueling the endothelial cell drive. Annu Rev Physiol.

[37] Rohlenova K (2020). Single-Cell RNA sequencing maps endothelial metabolic plasticity in pathological angiogenesis. Cell Metab.

[38] Hur J (2021). Sugar-sweetened beverage intake in adulthood and adolescence and risk of early-onset colorectal cancer among women. Gut.

[39] Zheng X (2021). Comprehensive assessment of diet quality and risk of precursors of early-onset colorectal cancer. J Natl Cancer Inst.

[40] Bu P (2018). Aldolase B-Mediated fructose metabolism drives metabolic reprogramming of colon cancer liver metastasis. Cell Metab.

[41] Stewart KL (2022). Association of sugar intake with inflammation- and angiogenesis-related biomarkers in newly diagnosed colorectal cancer patients. Nutr Cancer.

[42] de Heer EC, Jalving M, Harris AL (2020). HIFs, angiogenesis, and metabolism: elusive enemies in breast cancer. J Clin Invest.

[43] Wang Y (2018). New insights into the regulatory role of microRNA in tumor angiogenesis and clinical implications. Mol Cancer.

[44] Ntellas, P., et al., Old Player-New Tricks: Non Angiogenic Effects of the VEGF/VEGFR Pathway in Cancer. Cancers (Basel), 2020. 12(11). 10.3390/cancers12113145 PMC7692709 33121034

[45] Wang R (2020). B7–H3 promotes colorectal cancer angiogenesis through activating the NF-kappaB pathway to induce VEGFA expression. Cell Death Dis.

[46] Gomez-Zorita, S., et al., Comparative Effects of Pterostilbene and Its Parent Compound Resveratrol on Oxidative Stress and Inflammation in Steatohepatitis Induced by High-Fat High-Fructose Feeding. Antioxidants (Basel), 2020. 9(11). 10.3390/antiox9111042 PMC7690896 33114299

