# Supplementary material for: Fructose promotes angiogenesis by improving vascular endothelial cell function and upregulating VEGF expression in cancer cells
Source: J Exp Clin Cancer Res. 2023 Jul 28;42:184. doi: 10.1186/s13046-023-02765-3 (PMC10375648; doi:10.1186/s13046-023-02765-3)
Supplement: Supplementary file 1 — Additional file 1. Supplementary materials and Methods [file 13046_2023_2765_MOESM1_ESM.docx]

**Supplementary materials and Methods**

**Cell lines and cell culture**

Mouse colon cancer cell line (CT26.WT), human colon cancer cell line (SW620), human umbilical vein endothelial cell line (HUVEC) and HEK293T were obtained from American Type Culture Collection (ATCC; Manassas, Virginia, USA). Mouse lymph node endothelial cell line (SVEC4-10) and mouse colon cancer cell line (MC38) were obtained from Procell Life Science&Technology Co.,Ltd (Wuhan, China). CT26.WT, MC38 and SW620 cells were cultured in RPMI-1640 medium (Hyclone, South Logan, UT, USA). SVEC4-10 and HEK293T cells were cultured in DMEM (Hyclone). HUVEC cells were maintained in F12K medium (Gibco, Australia) containing 0.1 mg/mL heparin and 0.05 mg/mL endothelial cell growth supplement (ECGS). All media were supplemented with 10% fetal bovine serum (FBS, Hyclone). Dialysis fetal bovine serum (DFBS) was obtained by dialysis to eliminate glucose of the serum. Triple-free DMEM medium is sugarless, glutamine-free and pyruvic acid-free, prepared with triple-free DMEM powder. And it is required to add corresponding concentration reagent as utilizing. Hypoxia was defined as a 0.5% oxygen environment. The inhibitors used in the experiments were Glut5 inhibitor (2, 5-AM), HK2 inhibitor (2-DG), KHK inhibitor, Akt inhibitor (MK2206) and Src inhibitor (Dasatinib). The inhibitors were dissolved in DMSO and added to the cell culture medium at the corresponding action concentrations.

**Plasmid construction, lentivirus production and stable cell lines generation**

Full-length Glut5 was amplified by PCR using the following primers: upper: 5ʹ-ACCTCCATAGAAGATTCTAGAGCCACCATGGAGCAACAGGATCAGAGCA-3ʹ, lower: 5ʹ-TTCGAATTCGCTAGCTCTAGATCACTGTTCCGAAGTGACAGGTG-3ʹ, and cloned into the pCDH-CMV-MCS-Puro lentiviral vector at the XbaII cloning site. The construct was further confirmed by enzymatic digestion and DNA sequencing. Lentiviral production was achieved by a standard triple plasmid packaging system. Briefly, HEK293T cells were seeded into 10 cm culture dishes at 60% confluence 24 h prior to transfection, and then cotransfected with the lentiviral vector and two packaging plasmids using polyethyleneimine. After 48 h of transfection, the virus-containing supernatant was collected and used to infect cells. Stable cell lines were selected and maintained using 20 μg/mL puromycin.

**Cell Proliferation Assay**

Cell proliferation assays were performed according to the instructions of Cell Counting Kit-8 (CCK-8, Bimake, Houston, TX, USA). Briefly, cells were cultured in 96-well plates and treated as needed. At the indicated time points, CCK-8 solution (10 μL) was added to the medium and placed in a 37°C incubator for further incubation for 2-3 hours. The absorbance was measured at 450 nm to detect cell viability.

**Flow cytometry**

Cells were cultured in different concentration sugars for 24 h. These cells were harvested and fixed with 75% ethanol in 1.5 mL centrifuge tube overnight at 4°C, then centrifuged and washed twice with PBS. The cells were incubated with 50 μg/mL propidium iodide (PI) for 5~10 min at room temperature and detected by flow cytometry (BD, franklin lakes, New Jersey, USA). G0/G1, S and G2/M phase cell distributions were established using FlowJo_V10 software.

Cell apoptosis assays were performed according to the instructions of Annexin V-FITC/PI Apoptosis Assay Kit (Vazyme, China). In brief, cells were harvested and suspended with 300 μL binding buffer, then 5 μL Annexin V-FITC and 5 μL PI staining reagent were added for 10 min at room temperature. The stained cells were analyzed using flow cytometry (BD, franklin lakes, New Jersey, USA) FITC and PE passages. Apoptotic changes were analyzed using FlowJo_V10.

**EdU proliferation analysis**

Cell proliferation was determined by 5-Ethynyl-2’-deoxyuridine (EdU) assay kit (Cat#C0071S, Beyotime, China) following the manufacturer’s instructions. Briefly, 1 × 10^4^ cells/well were seeded in a 96-well plate for 24 h, and then the cells were cultured in medium with different sugars for 24 h. After that, these cells were incubated with 100 μl of 10 μM EdU per well for 2 h, and fixed with 4% paraformaldehyde for 30 min at room temperature. Subsequently, the cells were incubated with 2 mg/mL glycine for 5 min and permeabilization with 0.5% Triton X-100, and then reacted with a 1× Apollo solution for 30 min at room temperature in the dark. After that, the cells were incubated with 1× Hoechst solution for 30 min and then visualized by ﬂuorescence microscopy. EdU-labelled cells in each well were counted manually, and the percentages of EdU-positive cells were used to determine cell proliferative activity.

**Wound healing assay**

VECs were grown in 6-well plates until fusion, and wounds were prepared by scraping the cell monolayer using a 200 μL pipette tip. Simultaneously, medium containing different sugars was added to each well, and then wound widths were photographed using an inverted microscope at 0 and 16 h. Image J was used to calculate migration distances.

**Transwell assay**

Transwell analysis was performed using Boyden chambers with a pore size of 8 μm. To detect the cell migration ability, no sugar medium without FBS containing 6.0 × 10^4^ cells was placed in the upper chamber and the different conditioned medium containing 10% FBS was added to the lower chamber for 600 μL. After incubation for 6 h at 37°C, the migrated cells were fixed and stained. Finally, photographs were taken by microscope.

**Quantitative real-time PCR (qRT-PCR) analysis**

The detailed method for quantitative real-time PCR (qRT-PCR) was performed as described previously.[^1^](#_ENREF_1) Firstly, cells were lysed using Trizol reagent (Life Technologies, Carlsbad, CA, USA) to extract RNA, which was reverse transcribed into cDNA by HiScript II Q RT SuperMix (Vazyme, China). Finally, according to the manufacturer's protocol, qRT-PCR was performed using AceQ qPCR SYBR Green Master Mix (Vazyme, China). The expression of the gene was calculated by the method of 2^-ΔΔct and normalized with the β-actin hoses-keeping gene. The primer sequences required for the PCR experiments are supplemented in Extended Table 1.

**Western blot analysis**

After treatments, cells were lysed with SDS lysis buffer. Western blot was performed as described previously.[^1^](#_ENREF_1) Approximately 40 to 80 μg of protein of each simple was separated by SDS - polyacrylamide gel electrophoresis (PAGE) and then transferred onto polyvinylidene difluoride membranes (PVED). This was followed by milk blocking antigens and antibody incubation. The antibodies used in this study were: HK2 (Cat#sc- sc-130358, Santa Cruz, CA, USA), KHK (Cat#sc-377411, Santa Cruz), Glut5 (Cat#sc-271055, Santa Cruz), Src (Cat#2110, CST, MA, USA), p-Src (Cat#6943, CST) , Akt (Cat#9272, CST), p-Akt (Cat# 4056, CST), HIF1α (Cat#ab51608, Abcam, Cambridge, UK), FAK(Cat#13009S, CST), p-FAK(Cat #3281,CST), STAT3(Cat #12640, CST), p-STAT3(Cat #9134, CST), AMPK(Cat #2532, CST), p-AMPK(Cat#2535, CST), ERK(Cat#4695, CST), p-ERK(Cat#4370, CST), p38(Cat#9212, CST), p-p38(Cat#4511, CST) and β-actin (Cat#A1978, Sigma-Aldrich, MO, USA). The protein strip luminescence signal was then detected using the ECL kit (Millipore, Billerica, MA, USA) according to the manufacturer's protocol.

**Histological staining**

Briefly, the dissected tissues were fixed with 4% formaldehyde, and embedded in paraffin, and then 4 μm sections were prepared before histological staining. The sections were deparafffinized by xylene, dehydrated by gradient alcohol, and then stained with hematoxylin and eosin (H&E) using routine dyeing procedures. Meanwhile, antibodies of Glut5 (1:200), CD31 (Cat#ZA-0568, Zhongshan Company, Beijing, China), and VEGF-A (1:200, Cat#ab46154, abcam, MA, USA) were used for immunohistochemical (IHC) staining to detect their expression level in the tumor sections. In brief, the sections were deparafffinized and dehydrated, and then exposed antigen by high pressure heat repair and blocked endogenous peroxidase by 3% hydrogen peroxide solution. After that, the sections were incubated with primary antibodies overnight at 4°C, and incubated with secondary antibody for 1 h at 37°C. Subsequently, the sections were stained with diaminobenzidine (DAB) to visualize immunolabeling. Expression scores were calculated by multiplying the percentage and intensity fractions. The percentage scores were defined as follows: 1 (0%-25%), 2 (26%-50%), 3 (51%-75%) and 4 (76%-100%). Intensity scores were defined as follows: 0 (no staining), 1 (lower staining), 2 (moderate staining), and 3 (higher staining). All tissues were divided into high (score ≤ 6) and low (score > 6) groups according to the final score. For Glut5/CD31 double immunofluorescence staining and Ki67 (Cat#28074-1-AP, Proteintech Group, Chicago, IL) staining, the tissue sections after incubation of primary antibody were tested with fluorescently labeled secondary antibody, followed by nuclear staining with DAPI (4’,6-diamidino-2-phenylindole). Glut5/CD31 double IHC staining was performed according to the protocol of Double-stain IHC Kit (Cat#DS-0004, Zhongshan Company, Beijing, China).

**Enzyme-linked immunosorbent assays (ELISA)**

VEGF-A levels in the medium in which the cells were cultured for 24 h under different conditions were measured according to the instructions of Human VEGF-A (Vascular Endothelial Cell Growth Factor A) ELISA Kit (Elabscience Biotechnology Co.,Ltd). Briefly, a 96-well ELISA plate was incubated with the collected culture supernatant (100 μL/well) for 90 min at 37°C, and the wells were decanted followed by adding 100 μL Biotinylated Detection Ab working solution for 60 min at 37°C. After washing the wells three times, HPR Conjugate working solution (100 µL/well) was added and incubated for 30min at 37°C. Then, the wells were washed five times with washing buffer, and Substrate Reagent (90 µL/well) was added to the wells for 15 min at 37°C. Finally, after adding Stop Solution, the OD value was measured at 450 nm immediately.

**Intracellular ATP assay**

Intracellular level of ATP was measured using CellTiter-Glo^®^ Luminescent Cell Viability Assay kit (Promega, #7570) as our previously described.[^2^](#_ENREF_2) 1.5 × 10^5^ cells per well were seeded into 12-well plates, and the corresponding medium was added after the cells were attached, then intracellular ATP was measured at different times according to the experimental design. Briefly, 200 μL of CellTiter-Glo reagent was added to each well after the medium was removed, and the plates were placed on a shaker for 10 min to fully lyse the cells at room temperature. Luminescence reading was carried out with a SpectraMax M2 reader (Molecular Devices). According to the number of cells, the relative amount of ATP in each group was compared.

**References:**

1. Zhu Y, Zhang H, Han X, et al. STAT3 mediated upregulation of C-MET signaling acts as a compensatory survival mechanism upon EGFR family inhibition in chemoresistant breast cancer cells. Cancer Lett 2021;519:328-342.

2. Cui Y, Wang Y, Liu M, et al. Determination of glucose deficiency-induced cell death by mitochondrial ATP generation-driven proton homeostasis. J Mol Cell Biol 2017;9:395-408.

Supplemental Table 1 Sequence of primers used for qRT-PCR

| Name | Primer | Sequence | Length(bp) |
| --- | --- | --- | --- |
| Glut5  (Human)  VEGF  (Human)  VEGF  (Mouse)  β-actin  (Human)  β-actin  (Mouse)  bFGF  (Human)  bFGF  (Mouse)  ANG  (Human)  ANG  (Mouse)  PDGF  (Human)  PDGF  (Mouse)  HIF-1α  (Human)  HIF-1α  (Mouse) | Upper  Lower  Upper  Lower  Upper  Lower  Upper  Lower  Upper  Lower  Upper  Lower  Upper  Lower  Upper  Lower  Upper  Lower  Upper  Lower  Upper  Lower  Upper  Lower  Upper  Lower | 5’ CAAGGATGCCAACAGTGATGAAGAG 3’  5’ GCAGAGTCGCCACATCATTTGA 3’  5’ CCCTGATGAGATCGAGTACATCTT 3’  5’ AGCAAGGCCCACAGGGATTT 3’  5’ AGCGGAGCTCTGTCGCGAGA 3’  5’ GCACAGACTCCCGGGCTGG 3’  5’ CAGAGCAAGAGAGGCATCC 3’  5’ CTGGGGTGTTGAAGGTCTC 3’  5’ CCAGTTGGTAACAATGCCATGT 3’  5’ GGCTGTATTCCCCTCCATCG 3’  5’ GCTGTACTGCAAAAACGGGG 3’  5’ AGCCAGGTAACGGTTAGCAC 3’  5’ AGTAGTGCTTTCTGGGAGTGC 3’  5’ TTCTGTCCAGGTCCCGTTTT 3’  5’ AAAGCATCATGAGGAGACGGG 3’  5’ AACGTTTCTGAACCCCGCTG 3’  5’ CAAGCACACAGGAGGGTCTC 3’  5’ TCATCGAAGTGGACAGGCAAA 3’  5’ CTGCTGCTACCCTGCGTCT 3’  5’ GTCTTGCACTCGGCGATCAT 3’  5’ CGCACAGAGGTGTTCCAGAT 3’  5’ AGATGGGCTTCTTTCGCACA 3’  5’ ATCCATGTGACCATGAGGAAATG 3’  5’ TCGGCTAGTTAGGGTACACTTC 3’  5’ TCTCGGCGAAGCAAAGAGTC 3’  5’ AGCCATCTAGGGCTTTCAGATAA 3’ | 171  245  72  217  154  160  179  230  108  264  196  125  214 |
