# Supplementary figures and images for: Fructose promotes angiogenesis by improving vascular endothelial cell function and upregulating VEGF expression in cancer cells
Source: J Exp Clin Cancer Res. 2023 Jul 28;42:184. doi: 10.1186/s13046-023-02765-3 (PMC10375648; doi:10.1186/s13046-023-02765-3)

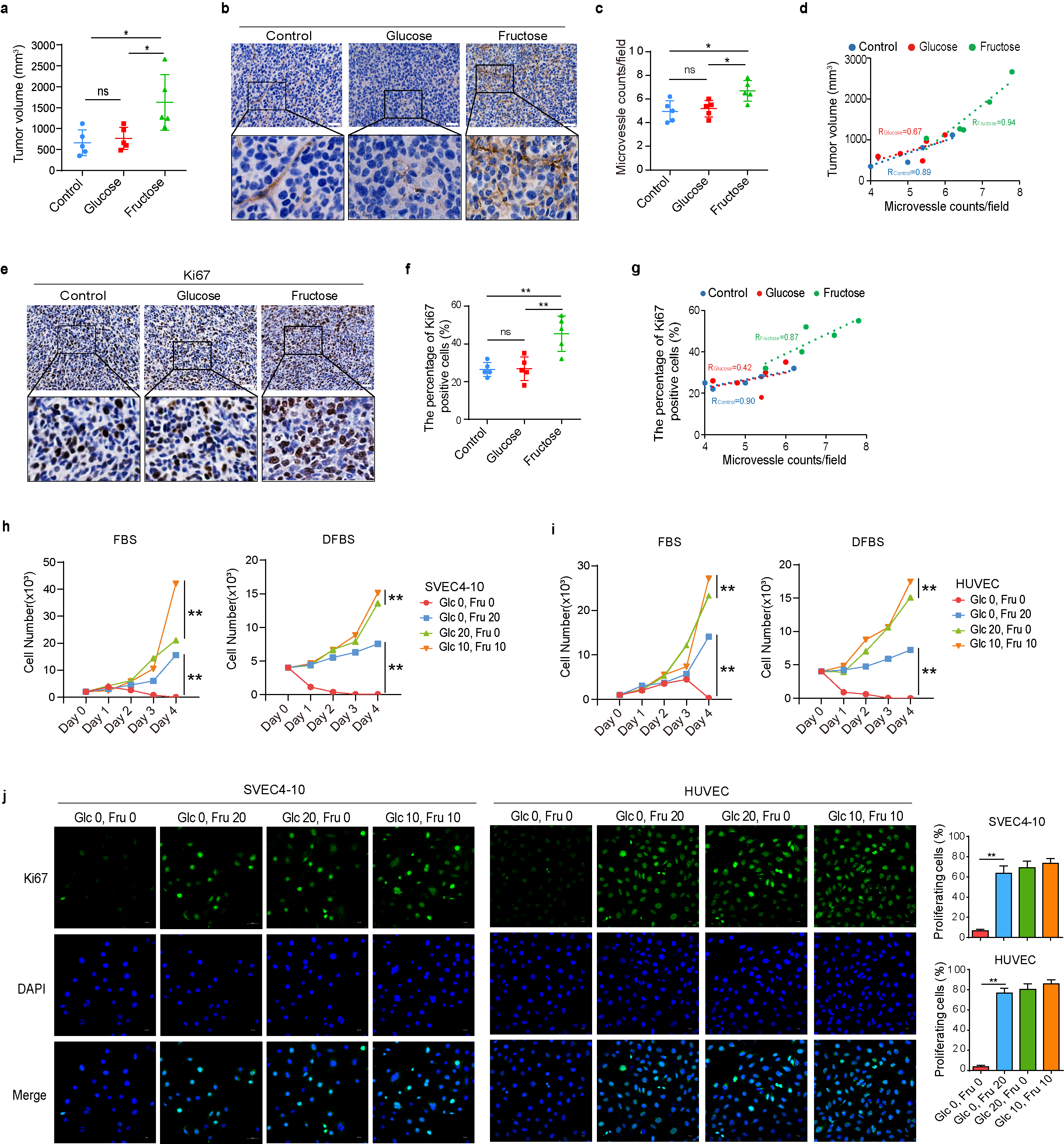

Supplement: Supplementary file 2 — Additional file 2: Fig. S1. Fructose promotes tumor angiogenesis in vivo and VEC viability in vitro. a, The volume of MC38 subcutaneous tumors in the indicated groups of mice (5 mice per group). b, Representative images of CD31 IHC staining of dissected tumors. Scale bar: 20 μm. c, MVDs counted by CD31 staining in dissected tumors. Scale bar: 20 μm. d, Correlation analysis between MVD and tumor volume. e, Ki67 positive index in tumor tissues of each group was detected by IHC staining. Scale bar: 20 μm. f, The percentages of Ki67-positive cells/all cancer cells were calculated. g, Correlation analysis between MVD and Ki‑67 proliferation index in tumors. h, Trypan blue staining was used to count the number of viable cells in different media to assess cell proliferation. [file 13046_2023_2765_MOESM2_ESM.tif]

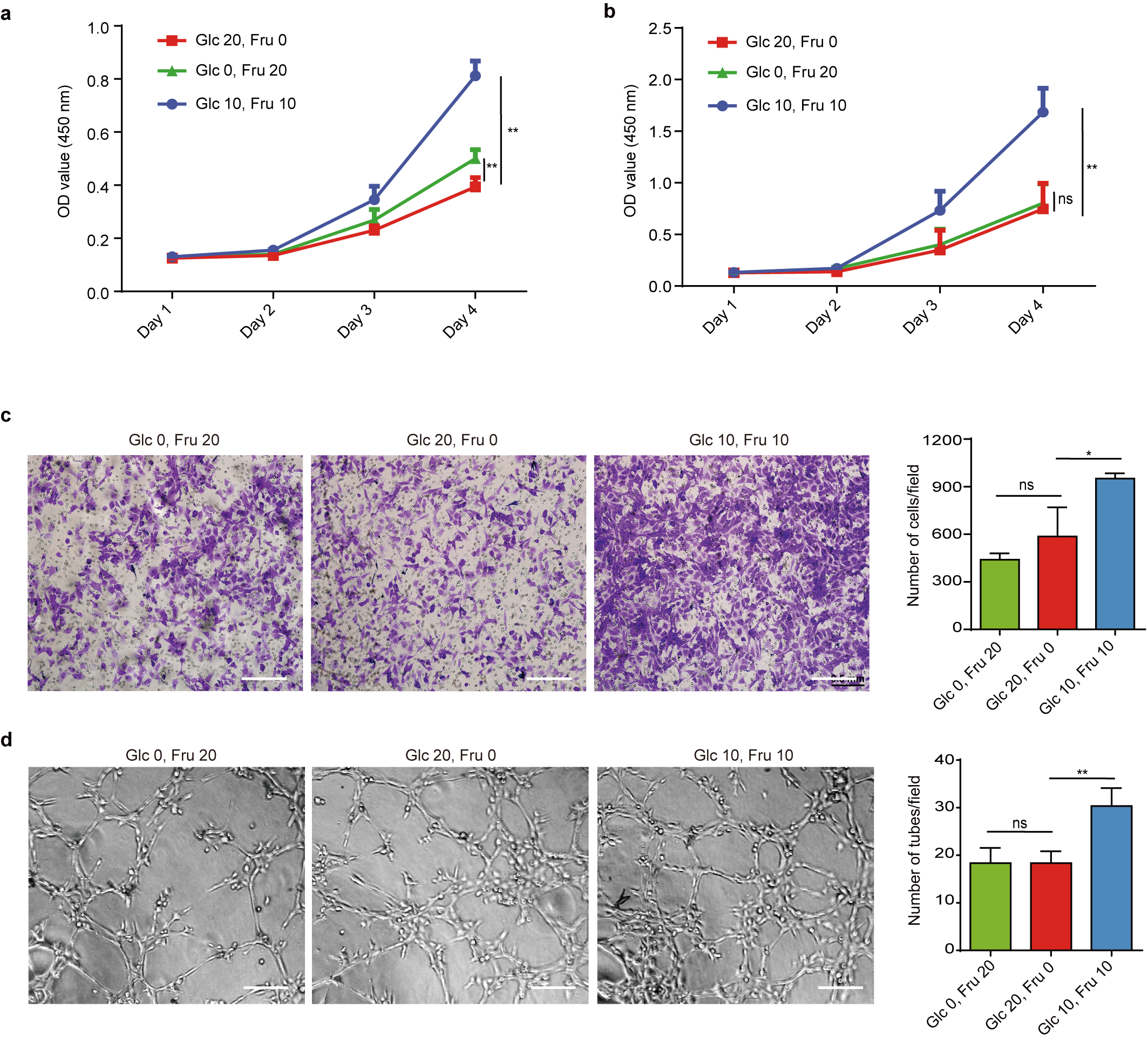

Supplement: Supplementary file 3 — Additional file 3: Fig. S2. Changes in the biological behaviors of VECs cultured in fructose medium for 2 weeks. a and b, The proliferation ability of SVEC4-10 cells in different media (Glc 0, Fru 20: 20 mM fructose only; Glc 20, Fru 0: 20 mM glucose only; Glc 10, Fru 10: 10 mM each of glucose and fructose) containing 10% DFBS (a) or FBS (b) after long-term fructose induction. c, The migration ability of fructose-induced SVEC4-10 cells in the different media. Scale bar: 100 μm. d, The tube-forming ability of fructose-induced SVEC4-10 cells in the different media. Scale bar: 50 μm. All data are expressed as the mean ± SD; ns, non-significant; *p < 0.05; **p < 0.01; n = 3. [file 13046_2023_2765_MOESM3_ESM.tif]

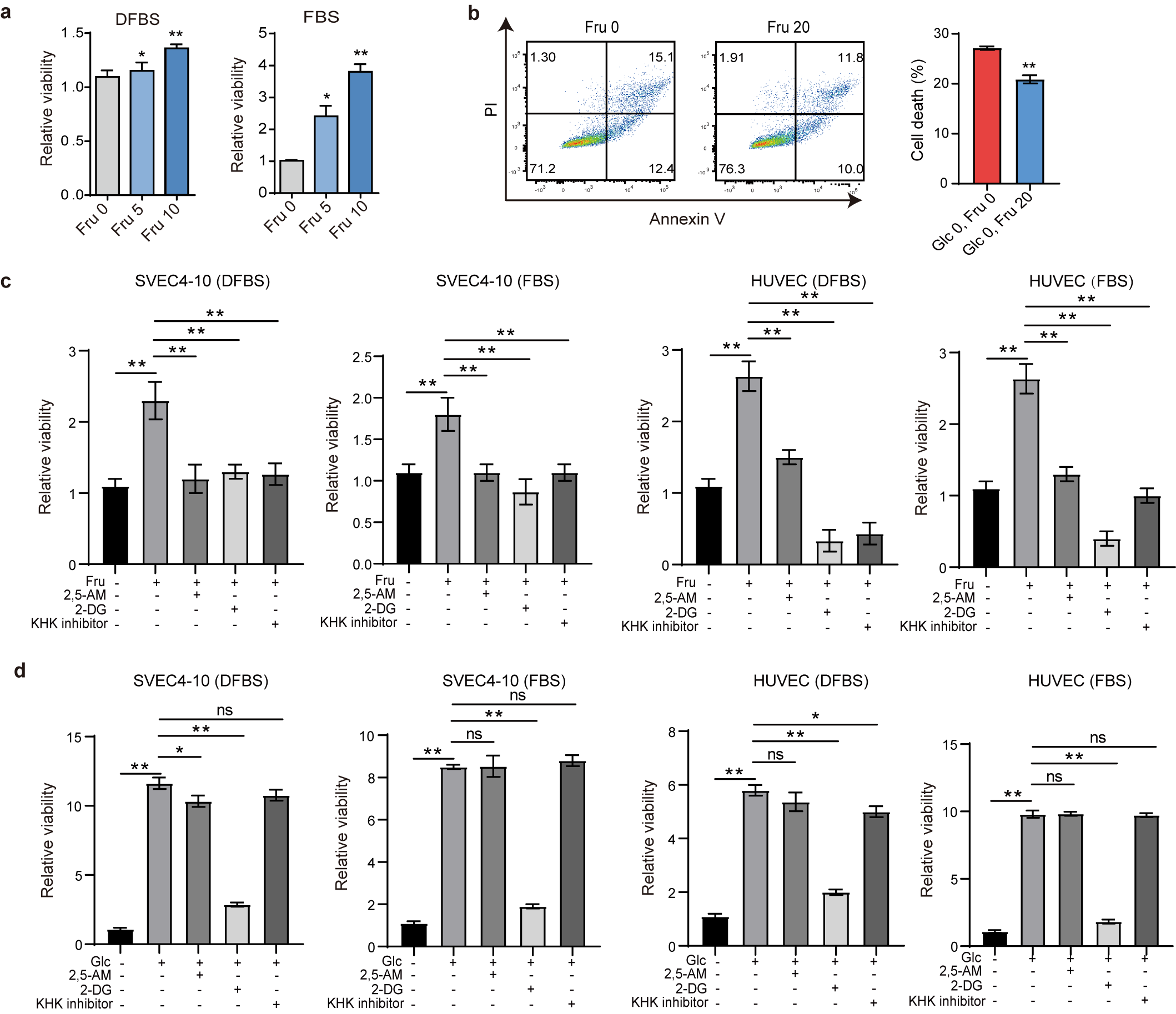

Supplement: Supplementary file 4 — Additional file 4: Fig. S3. Effects of fructose on VEC viability under hypoxic condition. a, CCK-8 analysis of the viability of SVEC4-10 cells cultured in glucose-free medium containing different concentrations of fructose, supplemented with 10% FBS or DFBS. b, Cell death analysis of SVEC4-10 cells cultured for 24 h in glucose-free medium with or without fructose (20mM). c, CCK-8 analysis of the viability of SVEC4-10 and HUVEC cells cultured in 20 mM fructose-containing medium in the presence of 2, 5-AM (3 mM), 2-DG (2 mM) or KHK inhibitor (1 μM) for 24 h under hypoxic condition. d, CCK-8 analysis of the viability of SVEC4-10 and HUVEC cells cultured in 20 mM glucose-containing medium in the presence of 2, 5-AM (3 mM), 2-DG (2 mM) or KHK inhibitor (1 μM) for 24 h under hypoxic condition. All data are expressed as the mean ± SD; ns, non-significant; *p < 0.05; **p < 0.01. n = 3. [file 13046_2023_2765_MOESM4_ESM.tif]

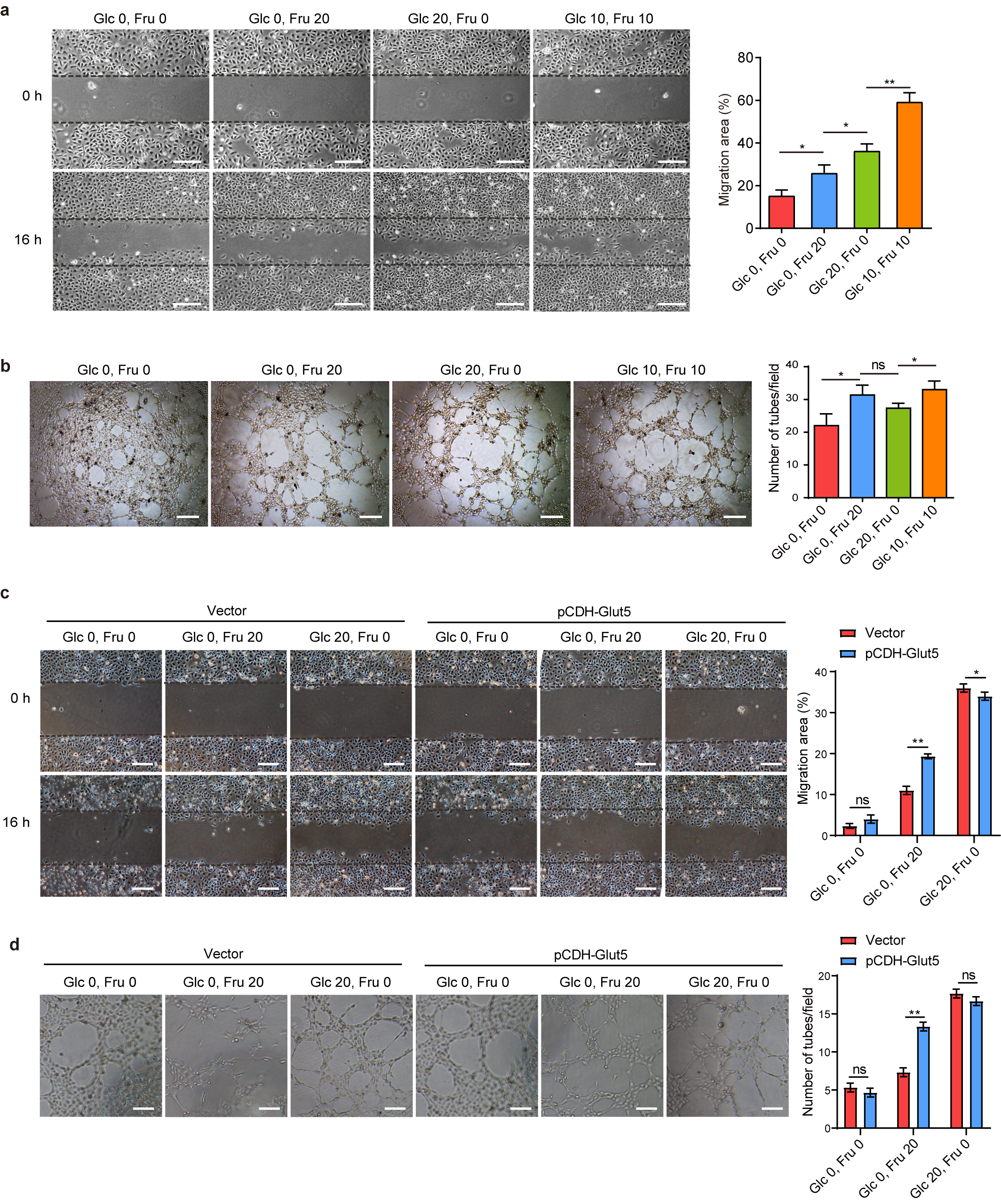

Supplement: Supplementary file 5 — Additional file 5: Fig. S4. Fructose promotes the migration and angiogenesis of HUVEC cells. a, The migration ability of HUVEC cells in four types of media (Glc 0, Fru 0: no glucose and fructose; Glc 0, Fru 20: 20 mM fructose only; Glc 20, Fru 0: 20 mM glucose only; Glc 10, Fru 10: 10 mM each of glucose and fructose) was analyzed by wound healing assay. Scale bar: 200 μm. b, Tube formation assay was used to analyze the angiogenic ability of HUVEC cells in four types of media. Scale bar: 50 μm. c and d, The migration (c) and tube-forming (d) abilities of HUVEC cells overexpressing Glut5 were assayed under different culture conditions. Scale bar: 200 μm (c) and 50 μm (d). All data are expressed as the mean ± SD; ns, non-significant; *p < 0.05; **p < 0.01; n = 3. [file 13046_2023_2765_MOESM5_ESM.tif]

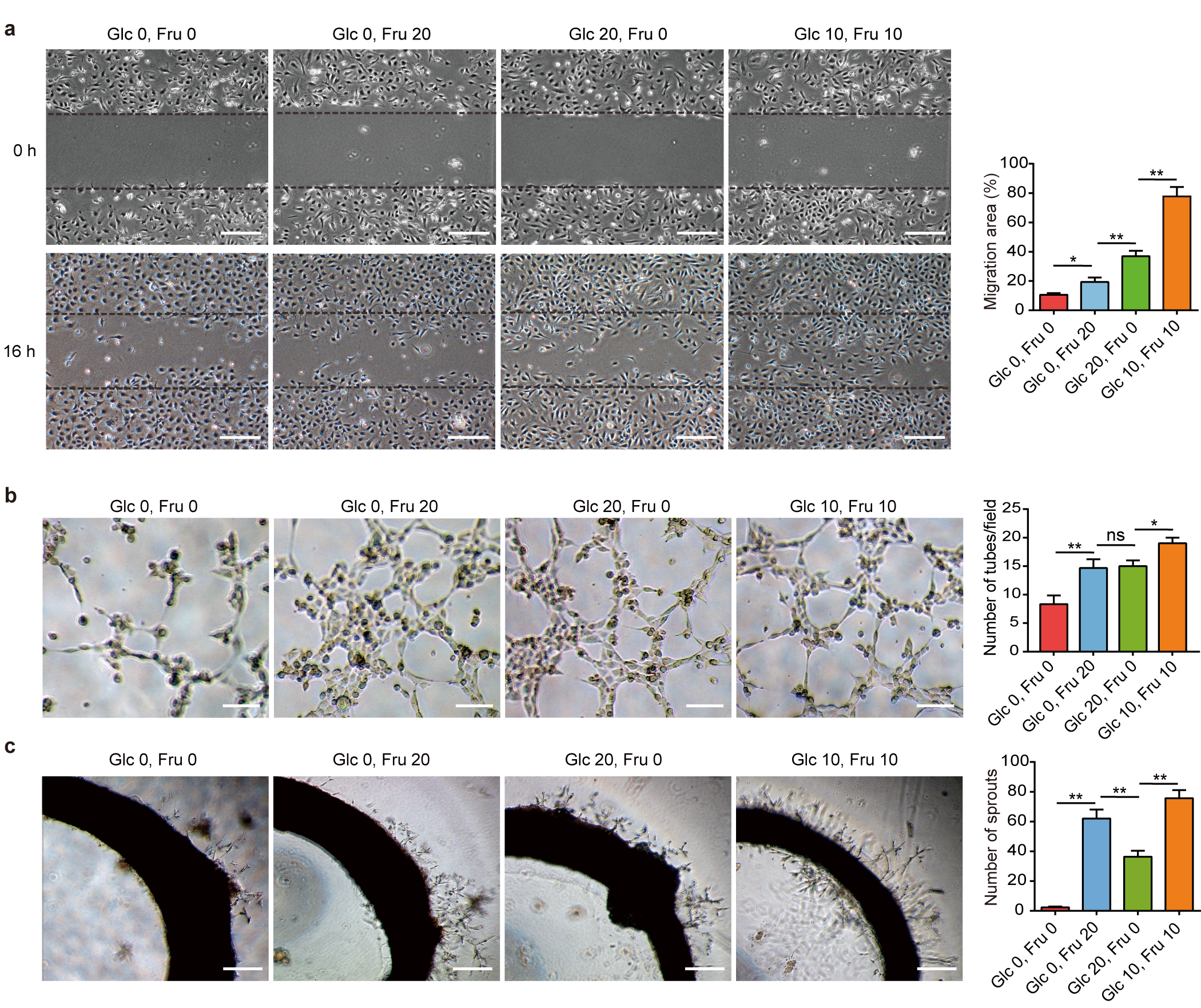

Supplement: Supplementary file 6 — Additional file 6: Fig. S5. Effects of fructose on biological functions of VECs under hypoxia. a, Wound healing assay was performed to analyze the migration ability of SVEC4-10 cells cultured in four different media under hypoxia for 16 h. Scale bar: 200 μm. b, The tube-forming ability of SVEC4-10 cells was analyzed under the indicated culture conditions under hypoxia for 4 h. Scale bar: 50 μm. c, The budding ability of rat aortic rings under the four culture conditions under hypoxia for 4 days. Scale bar: 500 μm. All data are expressed as the mean ± SD; ns, non-significant; *p < 0.05; **p < 0.01; n = 3. [file 13046_2023_2765_MOESM6_ESM.tif]

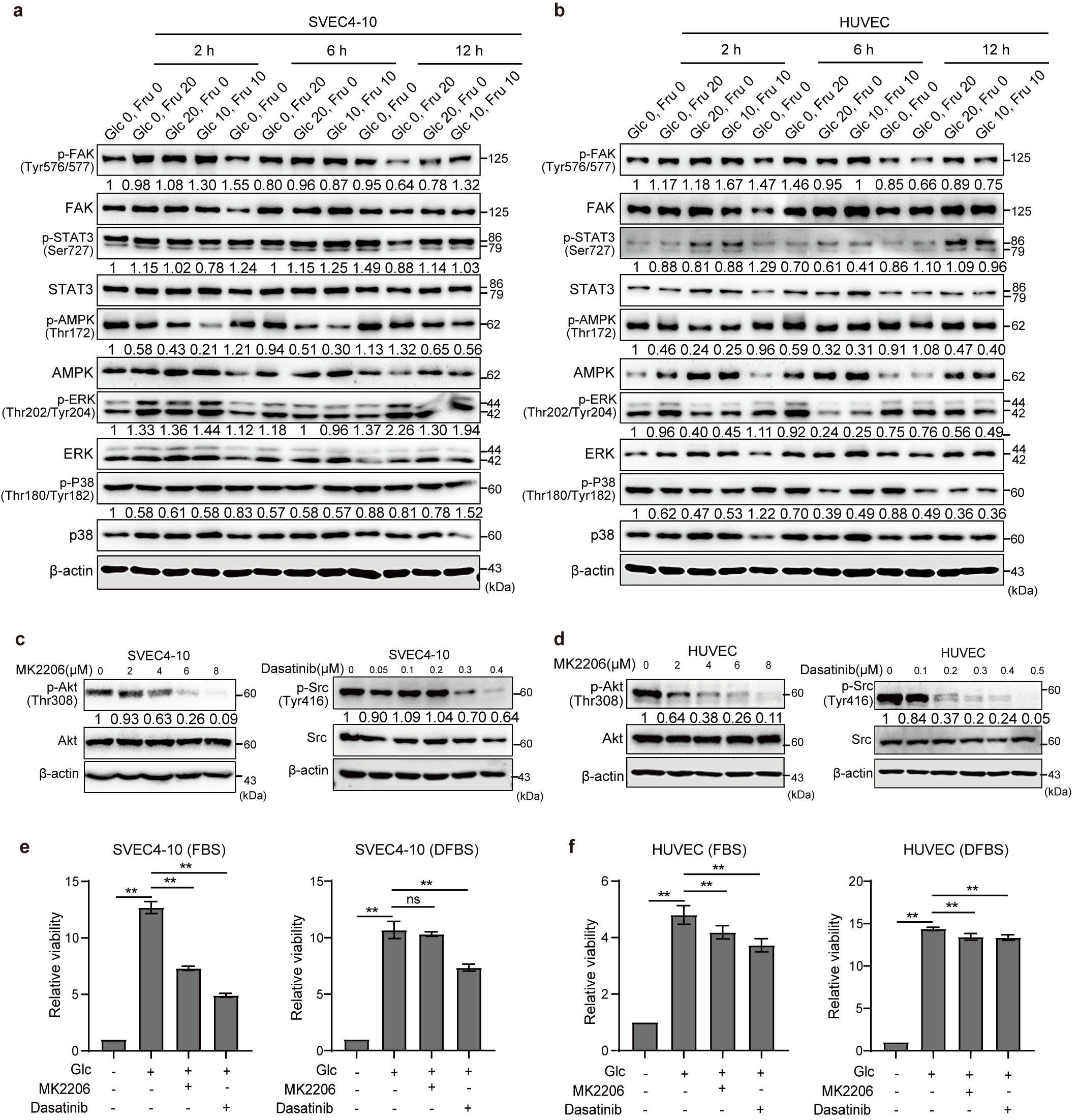

Supplement: Supplementary file 7 — Additional file 7: Fig. S6. Effect of fructose on key signaling pathways in VECs. a and b, SVEC4-10 (a) and HUVEC (b) cells were cultured in four types of media for 2 h, 6 h and 12 h, and then the activation of signaling pathways was detected by Western blot. Gray analysis was performed using Image J software, and the gray scale of each band was normalized to the mean value of that in the Glc 0, Fru 0 (2 h) group. c and d, Effects of different concentrations of MK2206 and Dasatinib on the activities of AKT and Src signaling pathways after treatment of cells for 2 h. Gray analysis was performed using Image J software, and the gray scale was normalized to the mean value of that in the no inhibitor group. e and f, CCK-8 assay was used to analyze the viability of SVEC4-10 (e) and HUVEC (f) cells cultured in 20 mM glucose medium with MK2206 (6 μM) or Dasatinib (0.3 μM) for 48 h. All data are expressed as the mean ± SD; ns, non-significant; *p < 0.05; **p < 0.01. n = 3. [file 13046_2023_2765_MOESM7_ESM.tif]

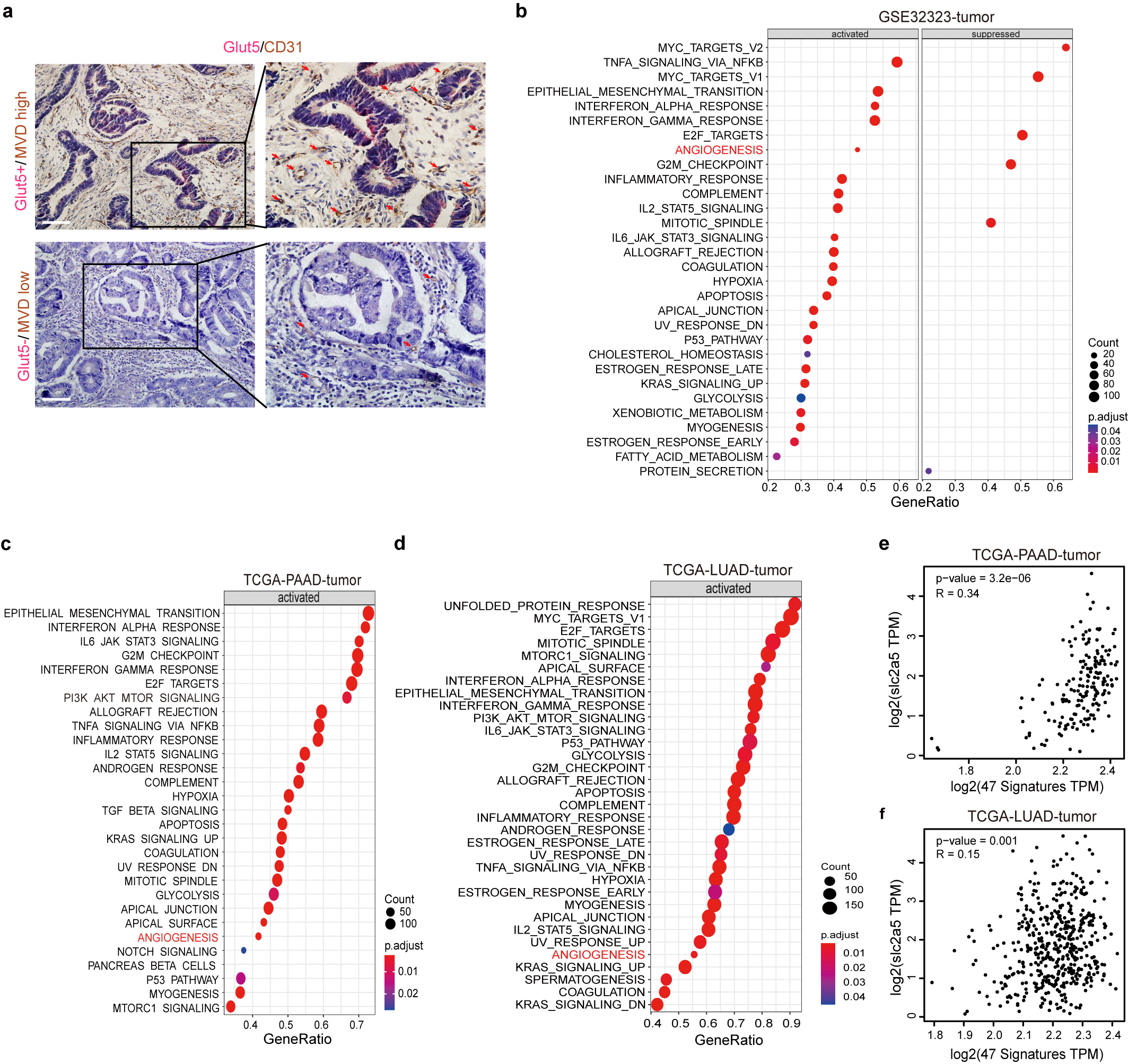

Supplement: Supplementary file 8 — Additional file 8: Fig. S7. Relationship between Glut5 mRNA expression levels and angiogenesis in tumor tissues. a, Representative images of double IHC staining of colorectal cancer tissues using Glut5 and CD31 antibodies. Glut5-positive cancer cells are stained red and CD31-stained microvasculars are stained brown, with red arrows indicating blood vessels. Scale bar: 50 μm. b, GSEA analysis identified up- and down-regulated pathways in tissues with high Glut5 mRNA expression based on the colorectal cancer dataset GSE32323. c and d, GSEA analysis identified up-regulated pathways in tissues with high Glut5 mRNA expression based on the TCGA dataset of pancreatic cancer (c) and lung adenocarcinoma (d). e and f, Correlation of 47 angiogenesis-related genes with Glut5 mRNA expression based on the TCGA dataset of pancreatic cancer (e) and lung adenocarcinoma (f). Analysis was performed using a two-tailed Pearson correlation analysis (e,f) [file 13046_2023_2765_MOESM8_ESM.tif]
